# Soft computing and statistical approach for sensitivity analysis of heat transfer through the hybrid nanoliquid film in rotating heat pipe

**DOI:** 10.1038/s41598-022-18736-1

**Published:** 2022-09-02

**Authors:** Ziya Uddin, Hamdy Hassan, Souad Harmand, Wubshet Ibrahim

**Affiliations:** 1 Department of Applied Sciences, SoET, BML Munjal University, Gurgaon, Haryana India; 2 Energy Resources Engineering Department, Egypt-Japan University of Science and Technology (E-JUST), Alexandria, Egypt; 3 LAMIH UMR CNRS 8201, Department de Mecanique, UPHF, Le Mont Houy, Valenciennes, France; 4 Department of Mathematics, Ambo University, Ambo, Ethiopia

**Keywords:** Mechanical engineering, Nanoscale materials, Theory and computation, Applied mathematics, Computational science, Computer science, Statistics

## Abstract

In this paper, the numerical solution for heat transfer through a rotating heat pipe is studied and a sensitivity analysis is presented by using statistical experimental design technique. Graphene oxide-molybdenum disulfide (GO-MoS_2_) hybrid nanofluid is taken as working fluid inside the pipe. The impact of the heat pipe parameters (rotation speed, initial mass, temperature difference) on the heat transfer and liquid film thickness is investigated. The mathematical model coupling the fluid mass flow rate and liquid film evolution equations in evaporator, adiabatic, and condenser zones of the heat pipe is constructed. The mathematical model is solved by implementation of “Particle Swarm Optimization” along with the finite difference method. The outcomes demonstrate that hybrid nanoparticles help to improve the heat transfer through the heat pipe and reduce liquid film thickness. The heat transfer rises with increasing temperature difference and reducing inlet mass, and it reduces slightly with rising rotation speed. The difference in liquid film thickness between the evaporator and condenser zones increases with increasing temperature difference and decreasing rotation speed. The impact of increasing the volume fraction of GO on the liquid film thickness is higher than that in the case of the MoS_2_ nanoparticles. However, an increase of the heat transfer is noticed in case of increasing the volume fraction of GO relative to increasing MoS_2_ concentration. Statistical analysis of the computed numerical data and the identification of significant parameters for total heat transfer are found using the response surface method. At 95% level of significance, the GO concentration in the hybrid nanofluid, inlet mass of the hybrid nanofluid and the temperature difference inside the evaporator zone of the pipe are found to be significant linear parameters for increasing heat transfer.

## Introduction

In every era of industrialization and technological advancement, the cooling technology has become an integrated part of the manufacturing and production. Thermal management is a very important part of almost every process, be it the cooling of industrial machinery, cooling of electronic components, cooling of computers and laptops, cooling of communication satellite or cooling of rotating motor etc. Due to heavy heat generation by continuously running motorized machines, the chances of wear and tear of the machines also become very high, therefore, it needs to be cool down simultaneously without stopping the machine. As in most of the engineering processes the machines have motorized rotating components, therefore the heat removal form such machines has received significant attention from decades. Gray^1^ invented the cylindrical heat pipe wipe with small taper at one side to remove the heat from rotating machines. The heat pipe is a metallic sealed hollow device which works on the principle of evaporation and condensation of a fluid utilizing the temperature of machine to be cooled. After this groundbreaking invention various researchers have performed the experimental and numerical studies to understand and further improve the heat transfer using heat pipes. Daniels et al.^2^ performed a mathematical and experimental study to analyse the heat transfer rates for different physical parameters and established a good concurrence between the experimental and theoretical results for Arcton 113. Tournier et al.^3^ developed a mathematical model to study the liquid and vapor flow along with the heat transfer rates as a function of time. They validated the results with available experimental data for heat pipe with water. Utilizing the natural and mixed convection in liquid film, a detailed heat transfer model for pipe with high speed rotations was developed by Song et al.^4^. Depending upon the use of the pipe the design of the heat pipe may vary. The details about different type of heat pipes, their working mechanism and numerous applications can be found in review paper by Faghri^5^. An innovative use of rotating heat pipes was suggested by Fasquelle et al.^6^. They used the small heat pipes to cool down the rotating motors by placing the heat pipes inside the motor gaps. These pipes rotate about the axis of the motor at high rotational speed. Song et al.^7^ Hassan et al.^8^ experimentally investigated the impact of distance of rotation axis from the axis of the pipe (radius of rotation) on heat transfer rates and found the reduction in temperature of heat pipe for increasing radii of rotation. Considering the spacecraft applications, an analytical form of solution for the trapezoidal grooved heat pipe was presented by Madhav et al.^9^. They also performed the experimental study and validated the proposed mathematical model. Shi et al.^10^ presented the simulations for rotating loop pipe for cooling the shaft of a motor and reported upto 36% improved heat transfer performance for the rotation speed of 10,000 rpm.

With the progression of innovation in the current era of industrial revolution and nanotechnology, the interest for better thermal management systems has expanded. The design and processes of these thermal management systems are being compact day by day. To further improve the cooling or heating rates the design of the thermal management system must be relooked, however because of multiple constraints this isn’t in reality truly attainable. Considering the applications of heat pipe, the alternative solution for this issue is to utilize the pipe fluid with improved physical and thermal properties. The customary coolant fluids like water, oil, alcohol et cetera have low conductivities, whereas, the metal and their oxides have high conductivities. It has been contemplated that the addition of a little amount of nano-sized particles of metal or their oxides into the liquid improves the properties of these liquids by a huge amount. These liquids are termed as nanoliquids. Due to the upgraded properties of nanoliquids the specialists in the field of heat pipe technology included nanoliquids into their investigations. Kang et al.^11^ performed an experimental study to analyze the effect of silver nanoparticle concentration and nanoparticle size on the thermal performance of cylindrical grooved pipe. Hassan et al.^12^ investigated the role of nanofluid in the vapor flow modelling of heat pipe. Bertossi et al.^13^ presented the numerical simulations for the heat transfer through cylindrical heat pipes. Venkatachalapathy et al.^14^ experimentally investigated the thermal resistance and heat transfer rates of a mesh wicked heat pipe with CuO-water nanofluid at different orientations of the cylindrical pipe. They reported a significant improvement in the thermal performance of the heat pipe with nanofluid. An analytical and experimental study on the use of Alumina-water nanofluid in cylindrical heat pipe with porous wick was presented by Ghanbarpour et al.^15^. They reported that the use of nanofluid is helpful in decreasing the entropy generation in the pipe. Uddin et al.^16^ presented a numerical study to predict the performance of rotating heat pipe with ethylene glycol based copper oxide nanofluid. They used the particle size dependent viscosity models and proposed a novel particle swarm optimization based technique to perform the numerical computations. It was concluded from the study that the nanofluid with smaller sized nanoparticles and higher concentration are helpful in improving the thermal performance of the heat pipe with high rotational speeds. An interesting numerical study focused on capillary evaporator of the heat pipe was performed by Boubaker et al.^17^. They analysed the effect of Alumina-water nanofluid and the position of evaporator grooves and concluded that the use of nanofluid in the pipe reduces the evaporator temperature significantly, and hence improves the heat transfer performance of the pipe. Ghorabaee et al.^18^ experimentally analyzed the performance of thermosyphon with water based alumina nanofluid and Triton X-100 mixture and reported approximately 43% reduction in the thermal resistance of the pipe for highest possible nanoparticle concentration. Shuoman et al.^19^ investigated the double tube cylindrical heat pipe with water based alumina nanoparticle of 40 nm size. They showed that the two-phased closed thermosyphons performed three times better with nanofluids as compared to the pure water. They also validated their experimental results with the numerical computations. An experimental study to analyze the heat transfer characteristics of gravity assisted cylindrical heat pipes with 1% alumnina nanoparticles in water was executed by Reji et al.^20^. They performed the experiments at various angels of inclination of the pipe and reported that using nanoparticles enhanced the performance by 41% and the maximum efficiency was recorded at 60 degree of inclination.

After the discovery of Graphene by the Nobel laureates Geim et al.^21^, another prolific field has opened up for huge thermal management applications. Specialists have demonstrated that that graphene is the lightest material with noteworthy heat conduction properties even at room temperature (Jauregui et al.^22^, Balandin^23^). Aside from this, graphene also has magnificent physical and chemical properties. As graphene is such an extraordinary material, therefore the graphene, its oxides and graphene nanofluids have received noteworthy attention in past decade. Esfahani et al.^24^ investigated the heat conduction property of water-graphine oxide nanofluid. They found a significant enhancement of heat conductivity with a small increase in nanoparticle concentration. Nazari et al.^25^ carried out the experimental study on the use of water based graphene oxide nanofluid in pulsating heat pipe. They reported a thermal resistance reduction of up to 42% with the increasing nanoparticle concentration. Experimental study on the effect of graphene-water nanofluid in circular grooved pipe was presented by Veerasamy et al.^26^. They also reported the increasing levels of graphene concentration improves the heat transfer performance of pipe.

After the disclosure of nanofluids with improved heat conduction properties, the nanofluids have gotten a critical consideration over the previous two decade. Aforementioned in the literature, numerous studies have proved naofluids to be a decent way to further enhance the performance of heat pipes. After these fruitful investigations, the scientists have additionally attempted for further improvement of the thermal and physical properties of nanofluids and synthesized the fluids by embedding two or more nanoparticles of different nature in the base fluid directly or in the form of composites and termed this new class of fluids as hybrid nanofluids. In the last decade many studies have shown that hybrid nanofluids may show promising results with respect to the enhanced heat transfer rates. Molybdenum Disulphide being the 2 dimensional materials with improved suspension stability (Su et al.^27^, Pham et al.^28^) has been used in the preparation of hybrid nanofluids. Hybrid nanofluids have been widely considered in various heat transfer related studies. Chu et al.^29^ carried out a numerical study to investigate the effect of nanoparticle shape on convection and flow of MoS_2_-GO-water nanofluid flowing through a cylindrical body. El-Gazar et al.^30^ presented a fractional model for solar cell performance using hybrid nanofluids. Heat pipe researchers have also evaluated the impact of hybrid nanofluids on its heat transfer performance. Ramachandran et al.^31^ carried out an experimental study to look into the cylindrical heat pipe performance using nanofluid with three different proportions of Al_2_O_3_ and CuO in water and reported the increased operating range of heat pipe in case of hybrid nanofluid having alumina and copper oxide concentrations in the ratio of 1:3. Bumataria et al.^32^ presented a nice survey on the use of hybrid nanofluids in heat pipes. Sözen et al.^33^ proposed the use of hybrid nanofluid loaded heat pipes to improve the efficiency of heat recovery systems. Bumataria et al.^34^carried out an experimental study to investigate performance of mesh wicked cylindrical pipe with CuO-ZnO-H2O hybrid nanofluid at different pipe orientation angles and concluded the best performance of the pipe at 60 degree inclination from horizontal direction. Zufar et al.^35^ investigated the factors affecting pulsating heat pipe efficiency. They considered water based alumina-copper oxide and silica-copper oxide hybrid nanofluids in their study and reported that the reduction in thermal resistance for silica-copper oxide–water nanofluid is more as compared to other considered fluids. Pandya et al.^36^ proposed a mathematical model to predict the heat transfer using a grooved cylindrical heat pipe with water based hybrid nanofluid. In this study, the groove parameters for optimum heat pipe performance were also suggested. Vidhya et al.^37^ prepared a ZnO-MgO hybrid nanofluid with blended water and ethylene glycol solution and reported the enhanced thermal properties of this hybrid nanofluid. They used the prepared nanofluid in wire meshed cylindrical heat pipe and observed an enhanced thermal performance up to 29%.

From the aforementioned survey, it is observed that numerous studies have been carried out to investigate the performance of heat pipes with mono and hybrid nanofluids for different configurations, and keeping various applications into consideration. Most of these studies were focused towards the experimental or numerical investigations on the use of nanofluids for improved performance of stationary pipes with meshes, pipe at different inclinations, and stationary thermosyphons etc., but rotating heat pipes have received very less attention. Apart from this, it is also observed that none of the numerical studies done so far has given any insight about the sensitivity of involved nanofluid and heat pipe parameters on the heat transfer performance of rotating heat pipe like the investigation done by Mehmood et al.^38^, where the authors utilized an statistical technique to fit a regression model for identifying the important parameters influencing the flow of a nanofluid over rotating disk. Recently, Li et al.^39^ presented a comprehensive and comparative review on the numerical and experimental studies of different types of rotating heat pipes. They also reported the same research gaps and proposed the utilization of nanofluids ought to be focused more in the future researches.

Therefore, the objective of the current work is to identify the significant factors responsible for heat transfer in the rotating heat pipe utilizing the most advanced ethylene glycol based GO-MoS_2_ hybrid nanofluids, and hence finding the sensitivity of these parameters on heat transfer. The proposed mathematical model couples the fluid mass flow rate and liquid film evolution equations in the three regions of the heat pipe namely evaporator, adiabatic, and condenser zones, which are solved by implementation of “Particle Swarm Optimization” along with the finite difference method. Finally, a numerical experiment is designed for response surface analysis of heat transfer with respect to involved parameters and the significant factors are identified. To the best of author’s knowledge, this type of investigation has not been performed by any author. The results presented in this problem identifies the important parameters controlling the heat transfer which have not been reported earlier. Hence, the presented study and the results are unique.

## Mathematical formulation

**Figure 1:**
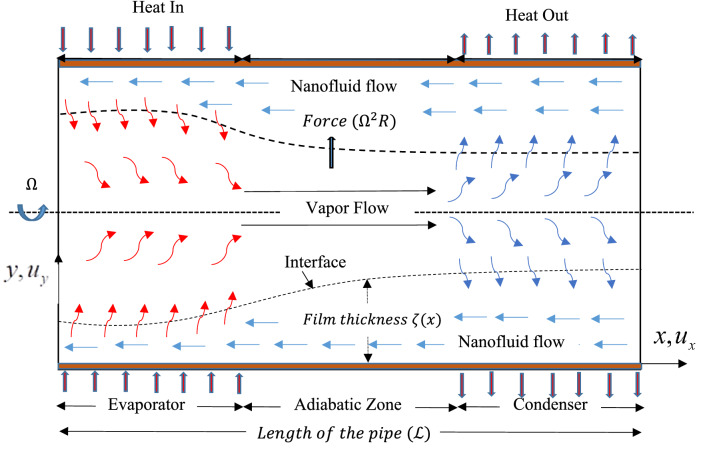
Physical Model and Coordinate system.

In the present mathematical model, the two dimensional laminar flow of hybrid nanoliquid is considered inside the thin film region, which is generated due to the high speed rotation of the cylindrical pipe. The x-axis is taken as the axis of the pipe which rotates about its own axis at a rate of $\Omega .$ Velocity $\left(\overrightarrow{V}\right)$ components in X and Y directions are ${u}_{x}$ and ${u}_{y}$ respectively. The physical mode of the pipe and the coordinate system are shown in Fig. 1.

Following Uddin et al.^16^ the equations for the flow of hybrid nanoliquid inside heat pipe are given as:

Continuity equation:

1 $$\overrightarrow{\nabla }.\overrightarrow{V}=0$$

Momentum equation:

2 $$\left(\overrightarrow{V}.\overrightarrow{\nabla }\right)\overrightarrow{V}=-\frac{1}{{\rho }_{hnl}}\overrightarrow{\nabla }P+\frac{{\mu }_{hnl}}{{\rho }_{hnl}}{\nabla }^{2}\overrightarrow{V}+\overrightarrow{F}$$

Due to the rotation of the pipe, centrifugal force ${\Omega }^{2}R$ acts against the gravitational force in Y-direction (Fig. 1). Therefore Eq. (1) and (2) in velocity components form are given as:

3 $$\frac{\partial {u}_{x}}{\partial x}+\frac{\partial {u}_{y}}{\partial y}=0$$

4 $${u}_{x}\frac{\partial {u}_{x}}{\partial x}+{v}_{y}\frac{\partial {u}_{x}}{\partial y}=-\frac{1}{{\rho }_{hnl}}\frac{\partial P}{\partial x}+\frac{{\mu }_{hnl}}{{\rho }_{hnl}}\left(\frac{{\partial }^{2}{u}_{x}}{\partial {x}^{2}}+\frac{{\partial }^{2}{u}_{x}}{\partial {y}^{2}}\right)$$

5 $${u}_{x}\frac{\partial {u}_{y}}{\partial x}+{v}_{y}\frac{\partial {u}_{y}}{\partial y}=-\frac{1}{{\rho }_{hnl}}\frac{\partial P}{\partial y}+\frac{{\mu }_{hnl}}{{\rho }_{hnl}}\left(\frac{{\partial }^{2}{u}_{y}}{\partial {x}^{2}}+\frac{{\partial }^{2}{u}_{y}}{\partial {y}^{2}}\right)+\left(g-{\Omega }^{2}R\right)$$

Practically, in rotating heat pipes the liquid flows from the condenser to the evaporator zone via adiabatic zone. In evaporator zone, the liquid film takes the heat from evaporator and the liquid gets evaporated. These vapors travel back to the condenser zone and gets condensed into the liquid again. In the present analysis velocity component ${u}_{y}$ is considered negligible. The inertia in the flow of hybrid nanoliquid is assumed very small as compared to other forces. The rate of linear mass flow is assumed to be zero at both the ends of the pipe and no slip condition is considered at the wall of the pipe. The hybrid nanoliquid film thickness $\zeta (x)$ is very small as compared to the radius of the pipe.

*Velocity and Mass flow boundary conditions*

6 $${\text{At extremities of the pipe}}:{\text{ at}}\;x = 0\;and\;x = {\mathcal{L}}\;{\text{Fluid mass flow rate}},\;\hat{M}_{hnl} = 0$$

7 $${\text{At }}\;{\text{the }}\;{\text{wall }}\;{\text{of}}\;{\text{ the}}\;{\text{ pipe}}:{\text{ at}}\;y = 0,\;u_{x} = 0\;\left( {{\text{No }}\;{\text{slip }}\;{\text{condition}}} \right)$$

At the boundary (liquid/vapor) of hybrid nanoliquid film (Daniels et al.^2^):

8 $$At\;y = \zeta \left( x \right), P_{liquid} = P_{vapor} = P_{sat} and \mu_{hnl} \frac{{\partial u_{x} }}{\partial y} = - \tau_{v} - \left( {\hat{\omega }_{vap} + u_{x, \zeta } } \right)\frac{{d\hat{M}_{hnl} }}{dx}$$

Here ${\widehat{M}}_{hnl}$ is the hybrid nanoliquid mass flow rate (Linear/per unit width) and given by

9 $${\widehat{M}}_{hnl}={\int }_{0}^{\zeta }{\rho }_{hnl}{u}_{x}dy$$

Using the above assumptions and boundary conditions (6)-(8), and following Uddin et al.^16^ the velocity the pressure term can be eliminated from Eqs. (4) and (5) and hence the velocity of the hybrid nanoliquid ($${u}_{x})$$ can be expressed as:

10 $${u}_{x}=-\frac{1}{{\mu }_{hnl}}\frac{\partial {P}_{\zeta }}{\partial x}\left(\frac{{y}^{2}}{2}-\zeta .y\right)-\frac{{\rho }_{hnl}}{{\mu }_{hnl}}\left({\Omega }^{2}R-g\right)\frac{\partial \zeta }{\partial x}\left(\frac{{y}^{2}}{2}-\zeta .y\right)-\frac{y}{{\mu }_{hnl}}{\tau }_{v}-\frac{y}{{\mu }_{hnl}}\frac{d{\widehat{M}}_{hnl}}{dx}\left({\widehat{\omega }}_{vap}+{u}_{x, \zeta }\right)$$

For high speed rotations the terms ${\tau }_{v}$ and ${P}_{\zeta }$ are negligibly small with respect to other terms (Song et al.^4^ also the vapor velocity $({\widehat{\omega }}_{vap})$ at the liquid/vapor boundary is very much larger than the nanoliquid velocity ${u}_{x, \zeta }$, therefore ${\widehat{\omega }}_{vap}+{u}_{x, \zeta }\approx {\widehat{\omega }}_{vap}$

Using Eq. (9) the per unit width of the film, the hybrid nanoliquid flow rate is given as:

11 $${\widehat{M}}_{hnl}=\frac{{\rho }_{hnl}^{2}}{{\mu }_{hnl}}\left(g-{\Omega }^{2}R\right)\frac{{\zeta }^{3}}{3}\left(\frac{\partial \zeta }{\partial x}\right)-\frac{{\rho }_{hnl}}{{\mu }_{hnl}}\frac{d{\widehat{M}}_{hnl}}{dx}{\widehat{\omega }}_{vap}\frac{{\zeta }^{2}}{2}$$

In hybrid nanoliquid film, the heat equation is written as:

12 $${u}_{x}\frac{\partial \theta }{\partial x}+{v}_{y}\frac{\partial \theta }{\partial y}=\frac{{k}_{hnl}}{{\rho }_{hnl}{Cp}_{hnl}}\left(\frac{{\partial }^{2}\theta }{\partial {x}^{2}}+\frac{{\partial }^{2}\theta }{\partial {y}^{2}}\right)$$

*Temperature boundary conditions*

13 $${\text{At }}\;{\text{extremities }}\;{\text{of}}\;{\text{ the}}\;{\text{ pipe}}: \, \;{\text{at}}\;x = 0\;{\text{and}}\;x = {\mathcal{L}},\;\theta = 0$$

14 $${\text{At}}\;{\text{ the}}\;{\text{ inner}}\;{\text{ wall }}\;{\text{of }}\;{\text{the}}\;{\text{ pipe}}:{\text{at}}\;y = 0,\;\theta = \theta_{w} \;and\;k_{hnl} \left( {\frac{\partial \theta }{{\partial y}}} \right) = H_{1} \left( x \right)$$

15 $${\text{At }}\;{\text{the }}\;{\text{outer }}\;{\text{wall }}\;{\text{of }}\;{\text{the}}\;{\text{ pipe}}: {\text{at}}\;y = \tau ,\;\theta = \theta_{w} \;and\;k_{Cu} \left( {\frac{\partial \theta }{{\partial y}}} \right) = H_{2} \left( x \right)$$

$\tau$ is the thickness of the pipe.

16 $${\text{At}}\;{\text{ the }}\;{\text{boundary}}\;{\text{ of}}\;{\text{ hybrid }}\;{\text{nanoliquid}}\;{\text{ film}}:{\text{ At}}\;y = \zeta \left( x \right),\;\theta = \theta_{s}$$

Here the condensor wall temperature is assumed to be constant over the length. It is also assumed that, the heat flow is only due to the condensation/evaporation of the hybrid nanoliquid film and the pipe wall in the direction perpendicular to the pipe axis.

Therefore, using Eq. (12) and the boundary conditions (13)-(16), the total heat flow per unit circumference of the pipe can be expressed as:

17 $$H\left(x\right)=\left({\theta }_{w}-{\theta }_{s}\right)/\left(\frac{\zeta \left(x\right)}{{k}_{hnl}}+\frac{\tau }{{k}_{Cu}}\right)$$

Following Daniels et al.^2^, $H\left(x\right)$ depends upon the average phase change enthalpy and given as

18 $$H\left(x\right)=-\widehat{\mathrm{\Delta h}}\frac{d{\widehat{M}}_{hnl}}{dx}$$

here $\widehat{\mathrm{\Delta h}}=\mathrm{\Delta h}+0.35Cp.\left(\Delta \theta \right)$ is the average enthalpy of vaporization.

Comparing Eqs. (17 and 18), gives

19 $$\frac{d{\widehat{M}}_{hnl}}{dx}=-\left({\theta }_{w}-{\theta }_{s}\right)/\widehat{\mathrm{\Delta h}}\left(\frac{\zeta \left(x\right)}{{k}_{hnl}}+\frac{\tau }{{k}_{Cu}}\right)$$

Heat input to the pipe through evaporator section is utilized in converting the liquid into vapor state, therefore

20 $$H\left(e\right)={\rho }_{v}\widehat{\mathrm{\Delta h}}{\widehat{\omega }}_{vap}$$

Using Eq. (17)

21 $$H\left(e\right)=H\left(x:0\le x\le {\mathcal{L}}_{e}\right)=\left({\theta }_{e}-{\theta }_{s}\right)/\left(\frac{\zeta \left(x\right)}{{k}_{hnl}}+\frac{\tau }{{k}_{Cu}}\right)$$

Combining Eqs. (19) and (20) we get:

22 $${\widehat{\omega }}_{vap}=\left({\theta }_{e}-{\theta }_{s}\right)/{\rho }_{v}\widehat{\mathrm{\Delta h}}\left(\frac{\zeta \left(x\right)}{{k}_{hnl}}+\frac{\tau }{{k}_{Cu}}\right)$$

Using Eq. (19) in (11) the hybrid nanoliquid film thickness change can be expressed as:

23 $$\frac{\partial \zeta }{\partial x}=\frac{3{\mu }_{hnl}{\widehat{M}}_{hnl}}{{\rho }_{hnl}^{2}\left(g-{\Omega }^{2}R\right){\zeta }^{3}}-\frac{3{\widehat{\omega }}_{vap}\left({\theta }_{w}-{\theta }_{s}\right)}{2{\rho }_{hnl}\left(g-{\Omega }^{2}R\right)\zeta \widehat{\Delta h}\left(\frac{\zeta }{{k}_{hnl}}+\frac{\tau }{{k}_{Cu}}\right)}$$

The net heat flux can be computed by using the formula: $$HeatFlux = 2\pi r(\Delta H)_{v} .\overset{\lower0.5em\hbox{$\smash{\scriptscriptstyle\frown}$}}{M}_{hnl}$$.

Here $(\Delta H)_{v}$ is enthalpy of vaporization.

Following Waini et al.^40^, the thermal and physical properties of hybrid nanoliquid are given by:

24 $${\text{Density}}:\;\rho_{hnl} = (1 - \phi_{2} )[\phi_{1} \rho_{p1} + (1 - \phi_{1} )\rho_{f} ] + \phi_{2} \rho_{p2}$$

25 $${\text{Specific heat capacity}}:\;(\rho C_{p} )_{hnl} = (1 - \phi_{2} )[\phi_{1} (\rho C_{p} )_{p1} + (1 - \phi_{1} )(\rho C_{p} )_{f} ] + \phi_{2} (\rho C_{p} )_{p2}$$

26 $${\text{Dynamic Viscosity}}: \;\mu_{hnl} = \frac{{\mu_{f} }}{{[(1 - \phi_{1} )(1 - \phi_{2} )]^{2.5} }}$$

*Thermal Conductivity*

27 $$k_{nl} = \frac{{k_{p1} + 2k_{f} - 2\phi_{1} (k_{f} - k_{p1} )}}{{k_{p1} + 2k_{f} + \phi_{1} (k_{f} - k_{p1} )}}k_{f} \;and\;k_{hnl} = \frac{{k_{p2} + 2k_{nl} - 2\phi_{2} (k_{nl} - k_{p2} )}}{{k_{p2} + 2k_{nl} + \phi_{2} (k_{nl} - k_{p2} )}}k_{nl}$$

where $\phi$ is nano-particle concentration in pure liquid, $\rho$ is the density and $C_{p}$ is the specific heat. The suffixes “p1”, “p2” and “f” are representing the GO-nanoparticle, MoS2-nanoparticle and pure fluid respectively.

Since the nano-particles are not considered in vapor phase, therefore the phase change enthalpy $\Delta H$ of the hybridnano-fluid will be due to the pure fluid only and given by the following relation:$(\rho \Delta H)_{hnl} = (1 - \phi_{hnl} )(\rho \Delta H)_{f}$, where $\phi_{hnl} = \phi_{1} + \phi_{2}$. The properties of the working fluid and nanoparticles are illustrated in Table1.

**Table 1:** Thermal and physical properties of GO, MoS_2_ and EG (Ziya et al. ^17^ and Chu et al. ^24^).

	GO	MoS_2_	EG
Density (kg/m^3^)	1800	5060	1105.2
Specific heat (J/kg.K)	717	397.21	2452.9
Thermal conductivity (W/m.K)	5000	904.4	0.2546

## Solution methodology

The Eq. (19) and (23) along with the boundary conditions (16–18) are solved in all the three sections of heat pipe in sequence. To solve the Eqs. (19) and (23) in all the three sections of the pipe the boundary conditions at the starting of each zone should be known. At the starting of evaporator zone (x = 0) the mass flow rate ${(\widehat{M}}_{hnl})$ is considered to be zero, but the liquid film thickness $\zeta (x=0)$ is unknown. Similarly to solve the equations in adiabatic and condenser zones the mass flow rates and liquid film thickness at the starting of each zone need to be known. Once $\zeta (x=0)$ is known the Eqs. (19) and (23) can be solved for the evaporator zone by assuming that the saturation temperature at the liquid–vapor interface is equal to 100 °C. The end of the evaporator zone is the starting of adiabatic zone $({\mathcal{L}}_{e}\le x\le ({\mathcal{L}}_{e}+{\mathcal{L}}_{a}))$, therefore the liquid film thickness and the mass flow rate at the end of evaporator zone are used as the required boundary conditions to solve the equations in adiabatic zone. In adiabatic zone the interface saturation temperature is considered to be same as the wall temperature of the pipe. The solution of this adiabatic zone would provide the required values to initiate the computation in condenser zone of the pipe. The inner wall temperature of the condenser zone is not known, which is to be computed. It is assumed that the mass flow rate at both the ends of the heat pipe is zero.

The total mass of the working fluid inside the heat pipe is utilized in the simultaneous liquid and vapor flow inside the pipe and for the efficient working of the pipe there is an optimal mass of the fluid which need to be inserted inside the pipe. Therefore apart from all the visible parameters in the questions (19) and (23) the mass of the fluid inserted inside the pipe is also considered as input parameter and the total heat transfer, liquid film profile and the condenser temperatures are estimated. To compute the outputs the “Particle Swarm Optimization” method is used in combination with the Runge–Kutta-Fehlberg scheme (see Uddin et al.^17^) to evaluate the unknown boundary conditions which are necessary to solve the system and hence the system of Eqs. (19) and (23) is solved.

In PSO the swarms are updated by using the following equations.

28 $$v\left[ {} \right] = \psi *v\left[ {} \right] + \phi_{p} *rand()*(Pbest\left[ {} \right] - x\left[ {} \right]) + \phi_{g} *rand()*(Gbest\left[ {} \right] - x\left[ {} \right])$$

29 $$x\left[ {} \right] = x\left[ {} \right] + v\left[ {} \right]$$

The overall objective of the proposed method is to determine the $\zeta (x=0)$ and Condensor temperature such that the following error function is minimized.

30 $$RRSS=\sqrt{{\left\{H(x=\mathcal{L})/H(x={\mathcal{L}}_{e})\right\}}^{2}+{\left\{{M}_{hnl}-{m}_{hnl})/{M}_{hnl}\right\}}^{2}}$$

Here RRSS represent the root of relative sum of squares.

${M}_{hnl}$=Average total mass. i.e. mass of liquid + mass of vapor inside the heat pipe.

${m}_{hnl}$ is the mass of the fluid inserted in the heat pipe to start the process.

**Figure 2:**
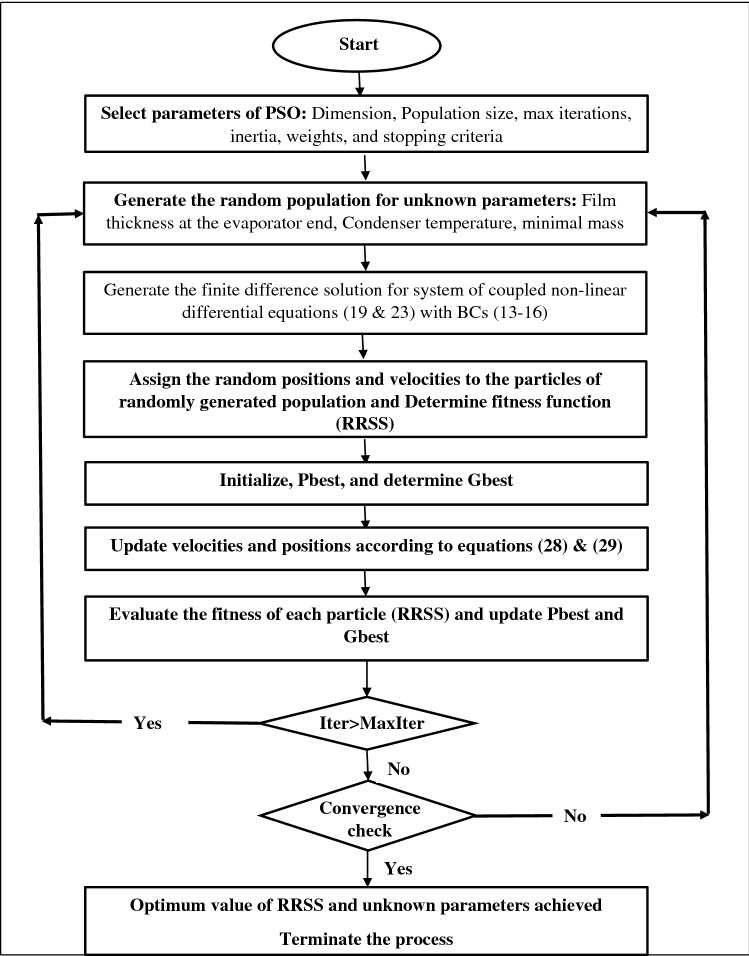
Flow chart of numerical algorithm.

The overall code has been developed to ensure that the heat input via evaporator zone will not be more than the heat taken out from the condenser zone and the liquid film thickness in evaporator zone is never larger that the film thickness at the end of condenser zone. The flow chart for the overall computation process is given below in Fig. 2.

## Validation of model

To validate our model computed results have been verified with experimental and simulated results available in the literature. For the special cases of the present model, the comparison of results are given below in Tables 2 and 3. From these tables it is observed that our results are in very good agreement with previously published experimental and theoretical studies. The results reported in the experimental studies and present computed results have a maximum variation up to 10% only, but there is no significant difference between the present results and the earlier published theoretical studies.

**Table 2:** Comparison of results for heat wipe containing 1 g of distilled water.

Evaporator length = 0.04 m, condenser length = 0.042 m, adiabatic length = 0.118 m, radius of pipe = 4 mm, evaporator temperature = 120 °C, rotation speed = 3000 rpm		
Parameters	Bertossi et al. ^13^	Present results
Average liquid film thickness	2.10 × 10^−4^ (m)	2.1103 × 10^−4^ (m)
Liquid film thickness at evaporator end	1.88 × 10^−4^ (m)	1.829 × 10^−4^ (m)
Liquid film thickness at condenser end	2.33 × 10^−4^ (m)	2.246 × 10^−4^ (m)
Total heat flux exchanged	73 (Watt)	72.8875 (Watt)

**Table 3:** Comparison of results with experimental and theoretical studies for different rotation speeds.

Comparison of results for heat wipe containing distilled water as inlet fluid Evaporator length = 121 mm, condenser length = 102 mm, adiabatic length = 184 mm, radius of pipe = 22.4 mm, thickness of pipe = 3 mm						
Evaporator Temperature (°C)	Condenser Temperature (°C) (Computed)	Temperature difference	Inlet mass (g)	Rotation speed (rpm)	Experimental study (Song et al. ^7^) Heat flow (Q) (Watts)	Present results Heat flow (Q) (Watts)
105	94.0516	10.9484	18.2	4000	99 (approx.)	96.9107
110	88.0690	21.931	18.2	4000	209 (approx.)	194.2134
115	82.0537	32.9463	18.2	4000	318 (approx.)	291.9484
120	76.0011	43.9989	18.2	4000	427 (approx.)	390.2330

Comparison of results for heat wipe containing EG as inlet fluid Evaporator length = 120 mm, condenser length = 120 mm, adiabatic length = 160 mm, radius of pipe = 10 mm						
Evaporator Temperature (°C)	Condenser Temperature (°C) (Computed)	Temperature difference	Inlet mass (g)	Rotation speed (rpm)	Ziya et al. ^16^ Heat flow (Q) (Watts)	Present results Heat flow (Q) (Watts
130	67.19	62.81	20.01	4181	137.9	137.9990
130	70.27	59.73	24.3	5091	104.8	104.8038
130	71.57	58.43	27.4	6000	90.7	90.7627
130	71.79	58.21	32.7	6000	74.8	74.7798
130	71.85	58.15	35.8	6000	67.6	67.5929

## Results and discussion

In this work, the impact of hybrid nanofluid and heat pipe parameters on the heat transfer and thin liquid film evolution are analyzed**.** The lengths of evaporator, condenser and adiabatic zones of the pipe are 120, 120 and 160 mm respectively. The inner radius and thickness the pipe are 10 and 1 mm respectively.

In these results the inlet mass (m_hnl_) changes from 18 to 24 gm, first nanoparticles (GO) volume fraction (ϕ_1_) from 0.01 to 0.04, second nanoparticles (MoS_2_) volume fraction (ϕ_2_) from 0.01 to 0.04, rotation speed (Ω) from 3 to 7 k rpm, temperature difference (ΔT) from 5 to 25 °C. During the variation of one parameter, the other parameters are fixed at m_hnl_ = 21 gm, ϕ_1_ = 0.02, ϕ_2_ = 0.02, ΔT = 20 C, and Ω = 5000 rpm.

### Impact of inlet mass

**Figure 3:**
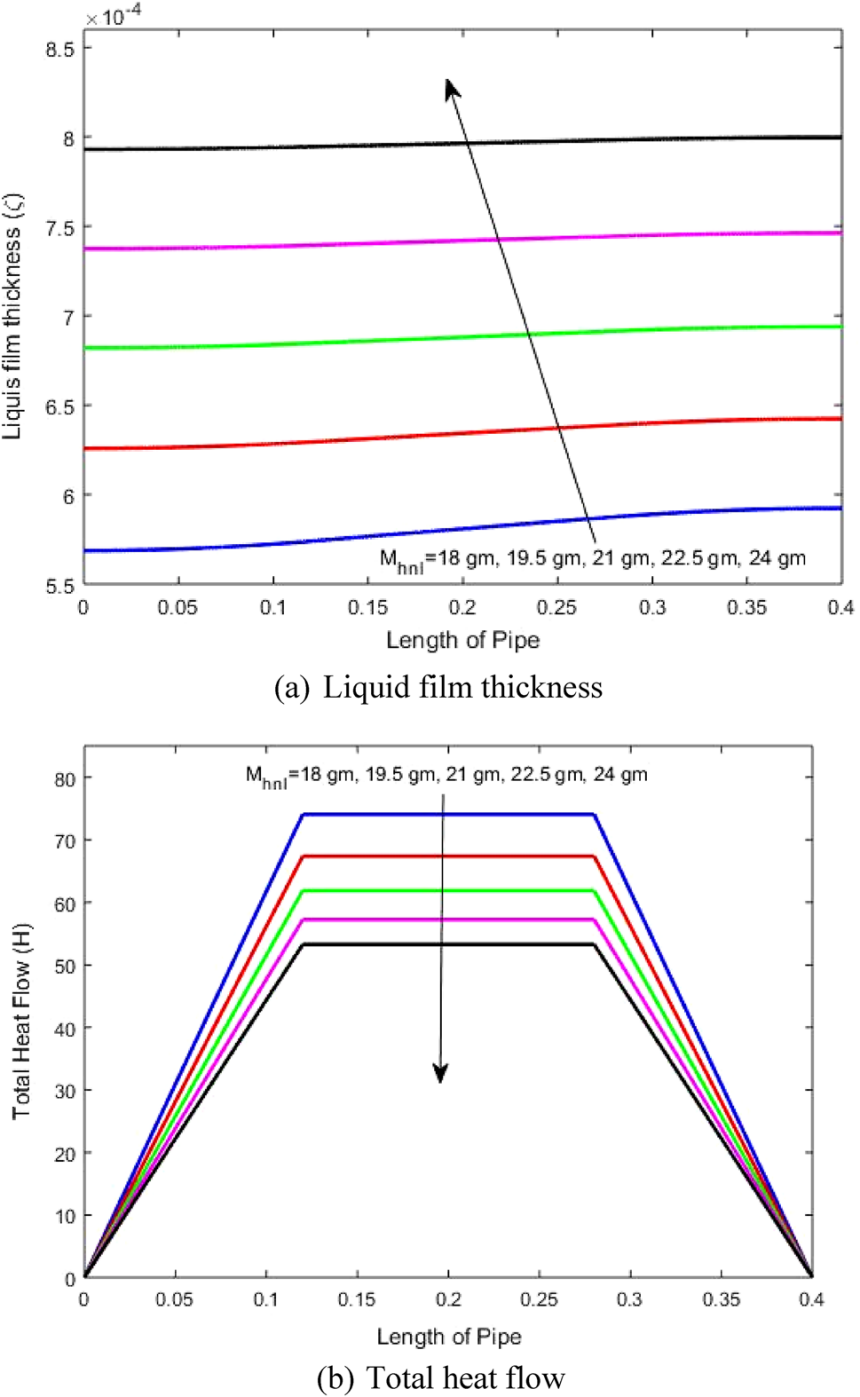
Impact of inlet mass on (**a**) liquid film thickness and (**b**) total heat transfer.

The impact of inlet fluid mass to the rotating heat pipe, on the evolution of thin liquid film and total heat transfer through the heat pipe length is illustrated in Figs. 3a, and b, respectively at ϕ_1_ = 0.02, ϕ_2_ = 0.02, Temp Diff (ΔT) = 20 C, and rotation speed (Ω) = 5000 rpm. Figure 3a reveals clearly that increasing the mass inlet to the heat pipe rises the liquid film thickness. This is due to that the evaporated mass as rising the inlet mass is not the same value of the rise in the inlet mass. Moreover, rising the inlet mass reduces the heat transfer rate and hence the evaporated mass and hence the liquid film thickness rises. It also shows that increasing the mass rises the liquid film thickness with approximately the same thickness throughout all the heat pipe length especially for higher inlet mass. Moreover, rising the inlet mass reduces the difference between the liquid film thickness between the condenser and evaporator because of reducing the evaporation rate of the liquid. It is found that rising the mass by about 33% rises the liquid film thickness by about 38%.

Figure 3b characterizes the rotating heat pipe into three zones, evaporator, adiabatic, and condenser. Through the evaporator, the heat transfer rises regularly throughout its length while the adiabatic zone is characterized by constant heat flux carried by the mass (the line is horizontal) and through the condenser zone, the heat transfer reduces until it becomes zero at the end of the condenser length. Moreover, this figure reveals that increasing the working fluid mass reduces the heat transfer. This is due to increasing the liquid film thickness as explained previous resulting rising of the thermal resistance of the liquid region and hence reducing the evaporation rate. Rising the mass by about 33% reduces the total heat transfer by about 31%. It is worth to indicate that as shown in Fig. 3b the impact of inlet mass on the heat transfer reduces with increasing this mass.

### Impact of first nanoparticles (GO) concentration

**Figure 4:**
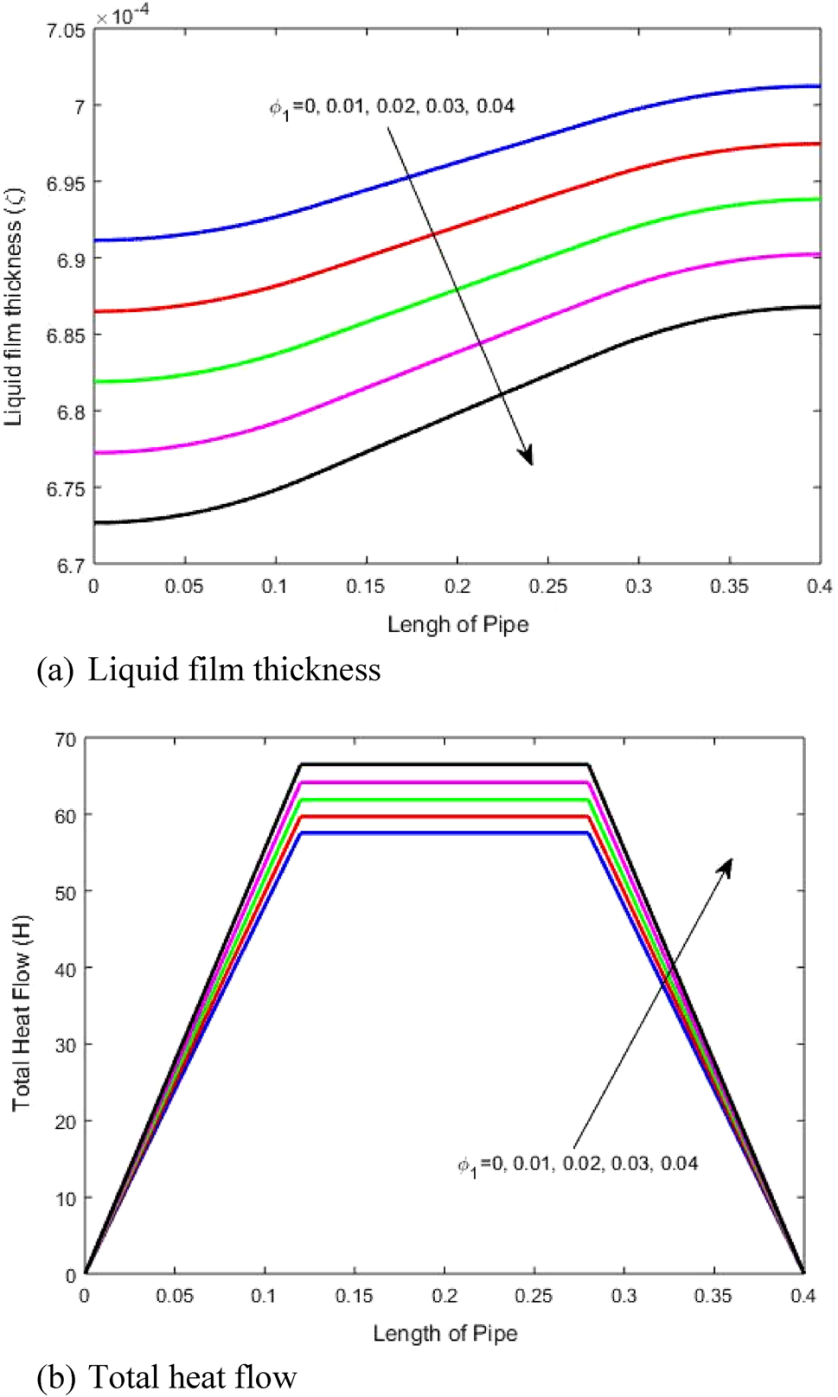
Impact of first nanoparticles (GO) volume fraction on (**a**) liquid film thickness and (**b**) total heat transfer.

The variation of the liquid film thickness and the total heat transfer with heat pipe length for different volume fractions of the first nanoparticles (GO) is illustrated in Figs. 4a and b respectively. Figure 4a illustrates that increasing the volume fraction of the nanoparticles reduces the liquid film thickness. This can be explained as follow; using nanoparticles with the working fluid of the heat pipe has multiple effects. Firstly, for an inlet mass to the heat pipe, the addition nanoparticles increases the heat pipe working fluid density for the nanoparticles with higher density than the working fluid. As the nanoparticles have higher density than the pure fluid (See Table 1), therefore nanofluid density increases with increasing volume fraction of the solid nanoparticles. However, the volume of the working fluid is nearly the same with and without using the nanoparticles, which rises the difference in liquid film thickness throughout the heat pipe length. Secondly, using nanoparticles rises the fluid thermal conductivity because the thermal conductivity of the nanoparticles is higher than that of the main fluid as mentioned in the Table 1. An increase in the thermal conductivity of the nanofluid rises the fluid evaporation resulting in a decrease of the liquid film thickness. It is clear that rising the nanoparticles volume fraction has almost the same effect on the liquid film thickness for the same percentage increase of the volume fraction. Rise in the working fluid thermal conductivity due to the use of highly conductive nanoparticles improves the evaporation as stated and hence rises the heat transfer through the heat pipe. Moreover, decreasing the liquid film thickness rises the heat transfer which explains the increase in heat transfer due to the nanoparticles as shown in Fig. 4b. Figure 4 indicates that rising the volume fraction of the nanoparticles from 0.01 to 0.04 reduces the liquid film thickness at the evaporator side from 7.02 × 10^−4^ m to 6.87 × 10^−4^ and increases the maximum total heat transfer from about 58 to 67 W. There is a significant improvement of approximately 16% is observed by increasing the nanoparticle concentration from 1 to 4%.

### Impact of second nanoparticles (MoS_2_) concentration

**Figure 5:**
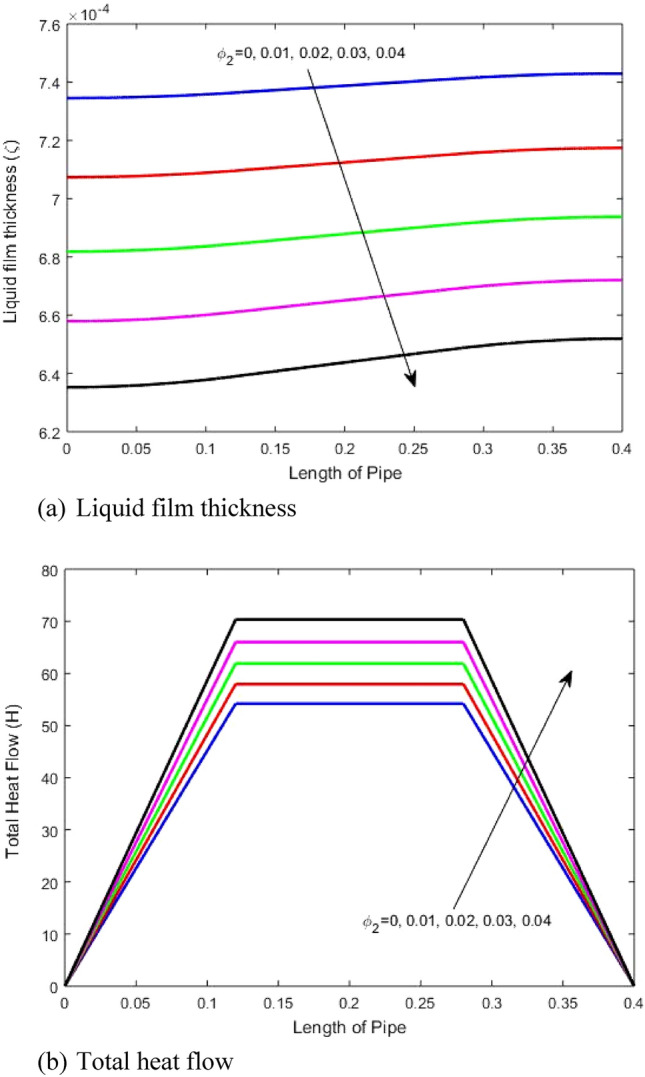
Impact of second nanoparticles volume fraction (MoS_2_) on (**a**) liquid film thickness and (**b**) total heat transfer.

The impact of the volume fraction of second nanoparticles (MoS_2_) on the thin liquid film evolution and total heat transfer through the heat pipe length is illustrated in Figs. 5a and b, respectively. The results show as stated previously that rising the liquid fraction of the nanoparticles reduces the liquid film thickness. This is due to that rising the liquid fraction of the nanoparticles rises the heat transfer within the liquid due to rising the thermal conductivity of the liquid as shown in Fig. 5b. Hence, this rises the evaporation rate of the liquid resulting a decrease of the liquid film thickness. If the results of the impact of the first (MoS_2_) and second nanoparticles (GO) on the liquid film thickness and total heat transfer are compared, it is found that the impact of the first nanoparticles on the liquid film thickness is higher than that of the case of the second nanoparticles because the thermal conductivity in case of GO is higher than that of MoS_2_ as stated in Table 1 which rises the evaporation of the liquid heat pipe working fluid. This result also shows an increase in the heat transfer in the case of GO relative to using MoS_2_. Moreover, it is found that in the case of using MoS_2_ the difference in liquid film thickness between the evaporator and condenser region is because of the higher density of the MoS_2_ compared to GO nanoparticles. The results show that the maximum increase due to rising the nanoparticles volume fraction from 0.01 to 0.04 of the liquid film thickness is about 1 × 10^−4^ and 0.18 × 10^−4^ m and total heat transfer is about 15 and 10 for GO and MoS_2_ nanoparticles, respectively.

### Impact of rotation speed

**Figure 6:**
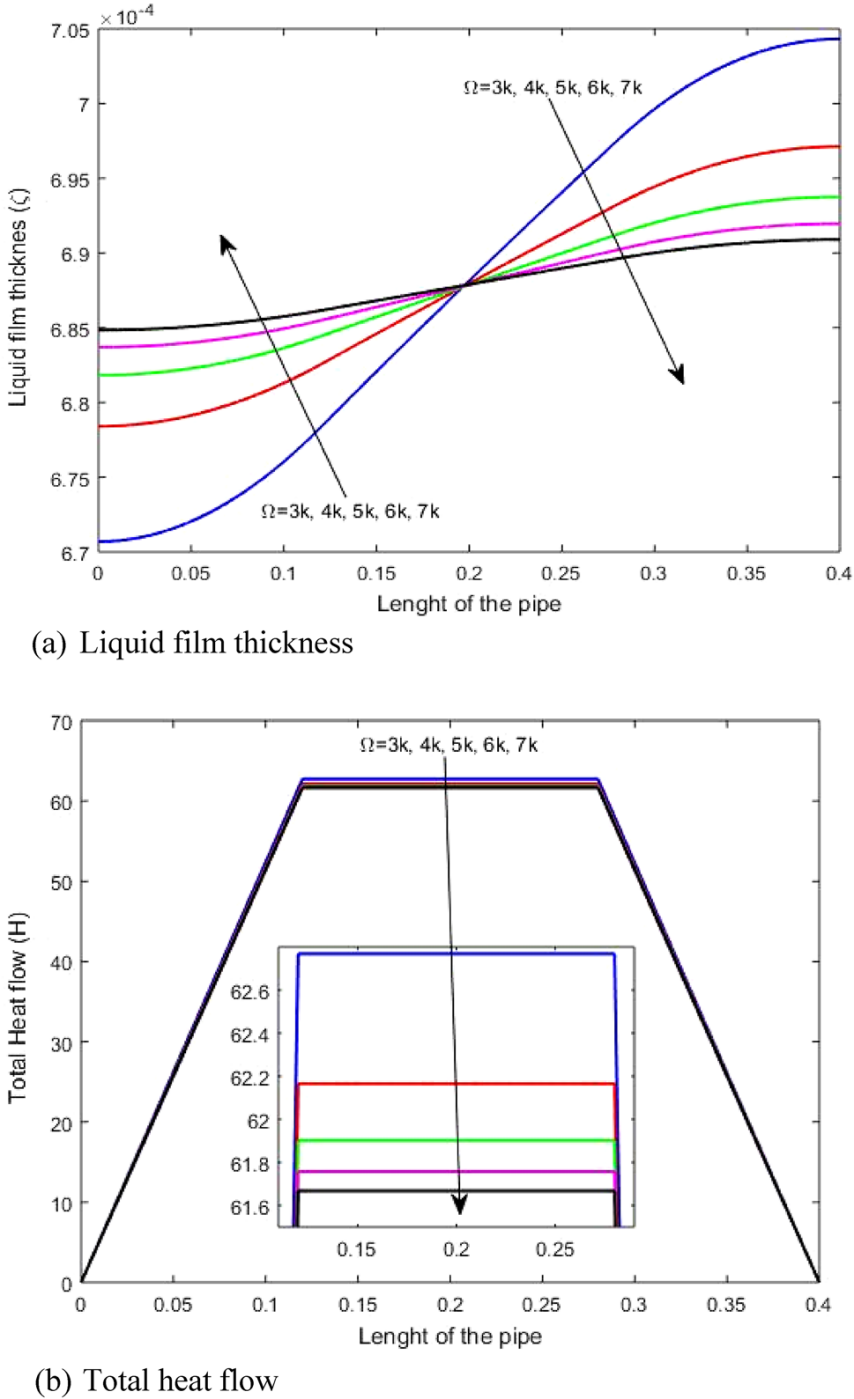
Impact of rotation speed on (**a**) liquid film thickness and (**b**) total heat transfer.

The impact of rotation speed on the liquid film thickness and total heat transfer through the heat pipe length is illustrated in Figs. 6a and b, respectively. It is clear that rising the rotation speed decreases the liquid film thickness between the evaporator and the condenser. This is due to the fact that, increasing the rotation speed yields the pressure difference between the condenser and evaporator section. So, this pressure difference through the heat pipe length permits the transfer of enough liquid flow. So, the difference of liquid film thickness between condenser and evaporator reduces due to increasing rotation speed. Hence, increase in rotation speed raises the liquid film thickness at the evaporator and reduces it at the condenser as shown in Fig. 6a. Figure 6b demonstrates that increase in rotation speed slightly reduces the heat transfer. It is noted that rising the rotation speed has negligible impact on the heat transfer within the heat pipe because the heat transfer proceed depends mainly on the working fluid properties.

### Impact of temperature difference

**Figure 7:**
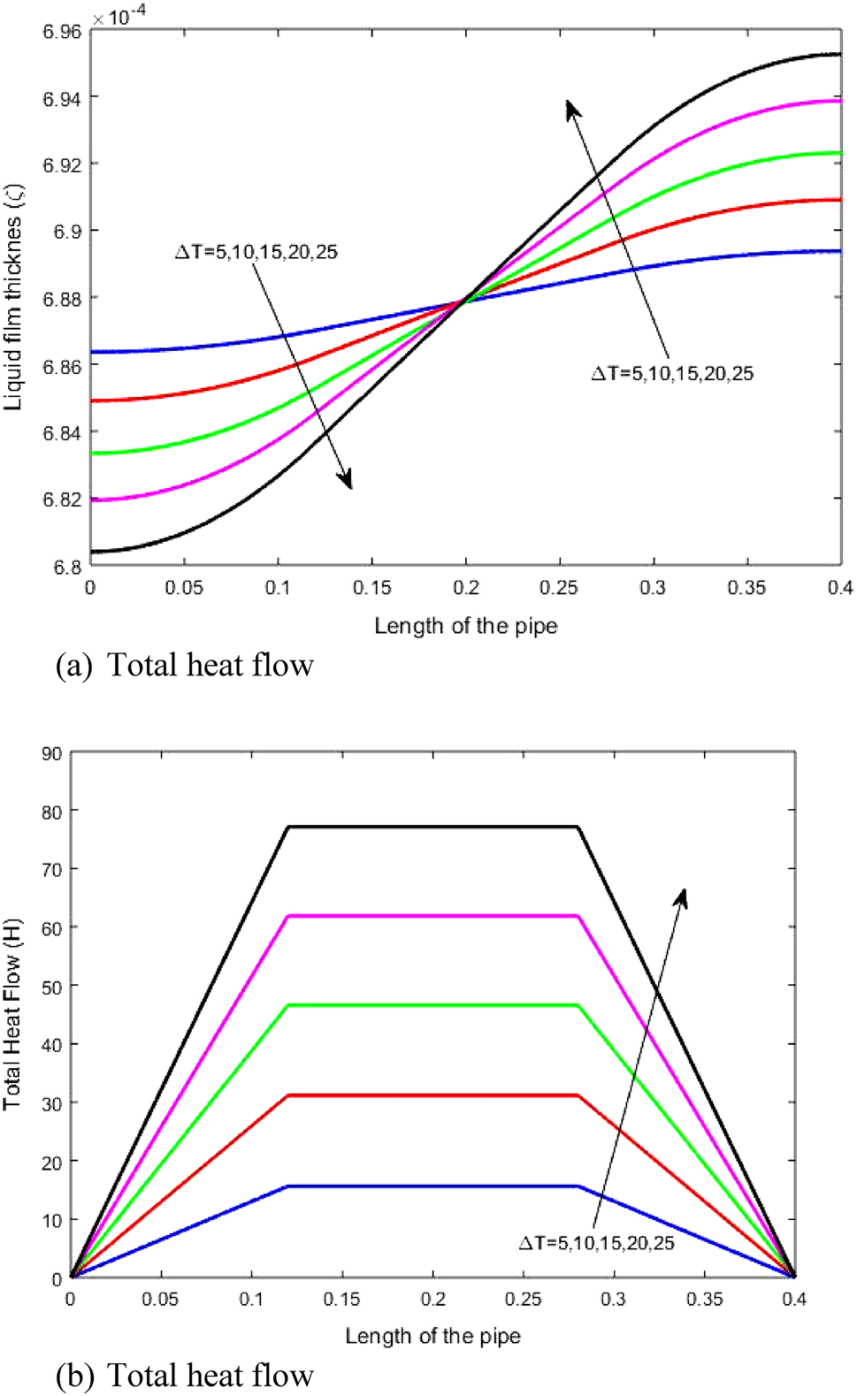
Impact of temperature differences on (**a**) liquid film thickness and (**b**) total heat transfer.

The impact of rising temperature difference (Δ*T)* between the evaporator and condenser on the liquid film thickness and total heat transfer through the heat pipe is illustrated in Figs. 7a and b, respectively. The figures reveal that when Δ*T* rises, the liquid film thickness, and the difference of the liquid film thickness of the condenser and evaporator rises. Also, rising *ΔT* increases the heat transfer through the heat pipe because it depends on the temperature difference between the evaporator and condenser zones. Rising the heat transfer increases the evaporated mass carrying the heat from the evaporator side to the condenser side. This mass condenses at the condenser side. Then, the condensed mass goes back to the evaporator side by a pressure difference (Δ*p*) between the condenser and evaporator. The increases of condensed mass leads to adjusting the thickness of the liquid film by rising the difference of the liquid film thicknesses between condenser and evaporator. This explains the cause of increasing the difference in the film thickness between the evaporator and condenser. Figure 7 reveals that increasing Δ*T* from 5 to 25 °C rises the difference in liquid film thickness from about 0.067 × 10^−4^ to about 0.145 × 10^−4^ and total heat transfer from about 15 to about 77.

## Statistical treatment

A statistical analysis is performed on the computed numerical results and a multi-regression fit and ANOVA is presented. The important factors which influence the heat transfer are identified using the sensitivity analysis. The details of the statistical treatments are explained in the below sub-sections.

### Response surface methodology (RSM)

Response Surface is a statistical method based on nonlinear regression fit. This method is used to optimize the output when two or more input variables are involved. The independent input variable or factors are called predictors and the output variable is known as response in this method. In RSM, a pre-defined combination (design of experiment) of input variables is used, and their responses to the output variable is optimize with the help of a quadratic model.

A second order regression model (full quadratic) is generally used in RSM. Considering “*f*” number of factors, the second order regression model can be written as:

31 $$Y={a}_{o}+\sum_{i=1}^{f}{a}_{i}{x}_{i}+\sum_{i=1}^{f}{a}_{ii}^{2}{x}_{i}^{2}+\sum_{i=1}^{f}\sum_{\begin{array}{c}j=1\\ i\ne j\end{array}}^{f}{a}_{ij}{x}_{i}{x}_{j}$$

Let the response and input variables are related by $Y=fun\left({x}_{i}, {x}_{j}\right)+ \in$

Where,$f$ and $\in$ represent the total no. of input variables (factors) and the noise or error in fitted quadratic model respectively. The surface generated by $Y=fun\left({x}_{i}, {x}_{j}\right)$ is called a response surface. For the best response surface fit, the method of least squares is used to minimize the error.

In the present problem, the Face Centered Central composite design is used to create the above said combination of considered input variables. In CCD, each input variable is set at a lowest, middle and highest values, where middle value of each variable is the average of lowest and highest values of the range of the corresponding variable. To make the whole process magnitude and dimension free theses values are coded as − 1, 0 and + 1 respectively, by using the formulae $c=2\left(\frac{var-{var}_{mid}}{{var}_{range}}\right)$ where $c$ represents the coded value, ${var}_{mid}=\left(\frac{{var}_{min}+{var}_{max}}{2}\right)$ and ${var}_{range}={var}_{max}-{var}_{min}$.

In face cantered CCD with half fractions, the total number of required combinations to generate the input/out data is given by formula $N={2}^{f-1}+2f+C$, where $f$ and $C$ are total number of input variables and number of points considered at the centre of design space respectively. In the present problem and $f$ is 5 and for face centred CCD the value of $C$ is 6. Therefore total number of experiments (numerical) is $N=32$. The coded combinations are given in the Table 4.

**Table 4:** Input output Table for CCD.

Nature of points	Numerical experiment Serial no	Coded Variable A (${\phi }_{{p}_{1}})$	Coded Variable B (${\phi }_{{p}_{2}})$	Coded Variable C ${(\widehat{M}}_{hnl})$	Coded Variable D $(\Omega )$	Coded Variable E $(\mathrm{\Delta T})$	Response/Output Y (Heat transferred, Q)
Corner points (${2}^{f-1}$)	1	− 1	− 1	− 1	− 1	1	67.2506
	2	1	− 1	− 1	− 1	− 1	36.1767
	3	− 1	1	− 1	− 1	− 1	38.4068
	4	1	1	− 1	− 1	1	83.3773
	5	− 1	− 1	1	− 1	− 1	24.3132
	6	1	− 1	1	− 1	1	51.8130
	7	− 1	1	1	− 1	1	54.9247
	8	1	1	1	− 1	− 1	29.7048
	9	− 1	− 1	− 1	1	− 1	33.4353
	10	1	− 1	− 1	1	1	71.4724
	11	− 1	1	− 1	1	1	75.8279
	12	1	1	− 1	1	− 1	40.8812
	13	− 1	− 1	1	1	1	48.1035
	14	1	− 1	1	1	− 1	26.0845
	15	− 1	1	1	1	− 1	27.6232
	16	1	1	1	1	1	58.7256
Axial points $(2f)$	17	− 2	0	0	0	0	43.3497
	18	2	0	0	0	0	50.0108
	19	0	− 2	0	0	0	40.8231
	20	0	2	0	0	0	52.9070
	21	0	0	− 2	0	0	69.7300
	22	0	0	2	0	0	35.2283
	23	0	0	0	− 2	0	47.0701
	24	0	0	0	2	0	46.4574
	25	0	0	0	0	− 2	15.6396
	26	0	0	0	0	2	77.0940
Repeated centre points $(C)$	27	0	0	0	0	0	46.5856
	28	0	0	0	0	0	46.5856
	29	0	0	0	0	0	46.5856
	30	0	0	0	0	0	46.5856
	31	0	0	0	0	0	46.5856
	32	0	0	0	0	0	46.5856

The input variable ranges viz. nanoparticle concentrations (${\phi }_{{p}_{1}}$ and ${\phi }_{{p}_{2}}$) in the base fluid, inlet nanofluid mass ${(\widehat{M}}_{hnl})$, the rotation speed of the pipe ($\Omega )$ and the temperature difference in the adiabatic and condenser zone of heat pipe ($\mathrm{\Delta T}$) are considered given below:

$0.01\le {\phi }_{{p}_{1}}\le 0.03,$ $0.01\le {\phi }_{{p}_{2}}\le 0.03,$ $18 gm\le {\widehat{M}}_{hnl}\le 24 gm,$ $4000\le\Omega \le 6000$ and temperature difference ${10}^{o} C\le \mathrm{\Delta T}\le {20}^{o} C$

### Developed mathematical model and ANOVA

**Figure 8:**
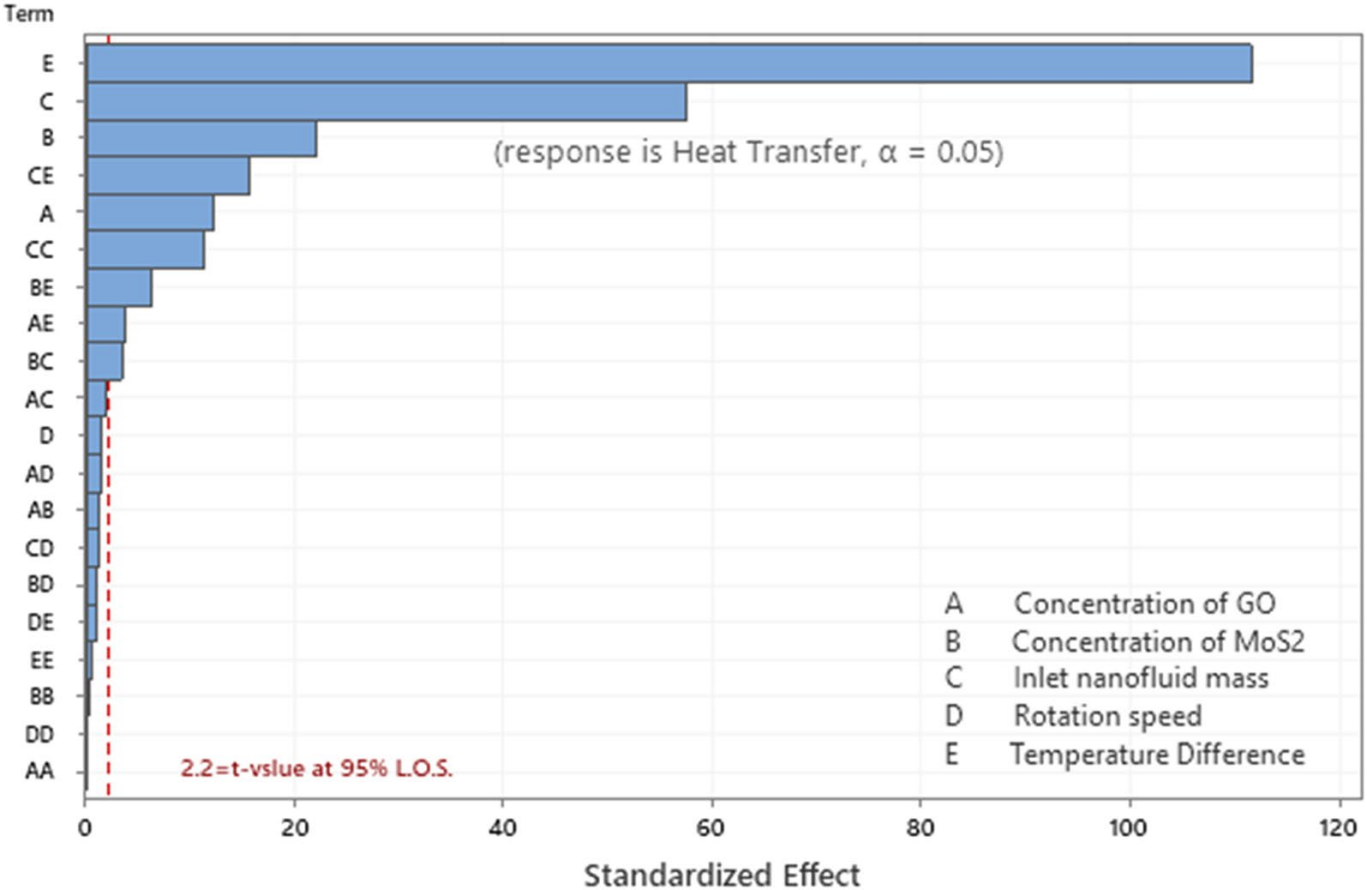
Pareto Chart for Heat transfer at 95% level of significance.

**Figure 9:**
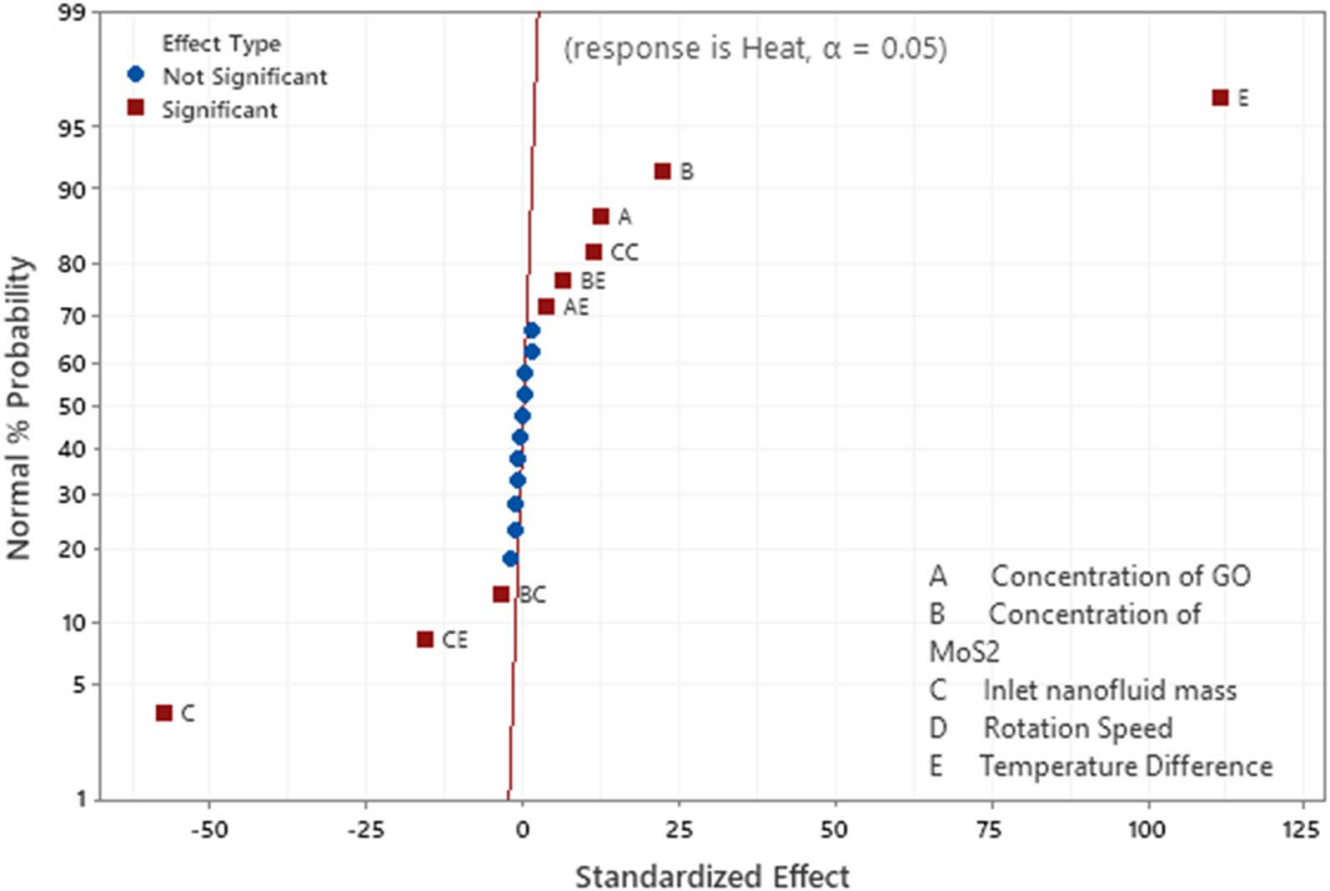
Normal plot for heat transfer.

The significant and insignificant input parameters and the interaction between the parameters are determined with the help of “Pareto chart at $\alpha =0.05$” shown in Fig. 8. The parameters/parameter interactions for which the magnitude of *t*-statistics values cross the threshold *t*-value (equal to 2.2) are identified as significant parameters otherwise insignificant parameters. The normal plot in Fig. 9 also help in the identification of significant or insignificant parameters. The parameters away from the straight line are marked as the parameters or two-parameter interactions that are significantly responsible for the heat transfer from evaporator to the condenser via the adiabatic zone of the heat pipe. Based on the analysis and adopting the above process, the resultant quadratic regression equation for the significant parameters and significant parameter interactions in coded units is given in Eqs. (32).

Mathematical model in coded form

32 $$Q=46.610 + 1.736\mathrm{ A }+ 3.125\mathrm{ B }- 8.106\mathrm{ C }+ 15.741\mathrm{ E}+ 1.449 {C}^{2} + 0.638\mathrm{ AE}- 0.593\mathrm{ BC}+1.101\mathrm{ BE }- 2.699\mathrm{ CE}$$

### Analysis of variance

Analysis of variance is also performed and presented in the Table 5. The statistical significance of the terms in regression equation are determined by computing the *p*-values using F-distribution. As, $\alpha =0.05$ is used for this purpose, therefore, the terms with *p*-value (probabilities) less than 0.05 are mentioned as statistically significant terms and the terms correspond to *p*-value more than 0.05 are notified as statistically insignificant terms. Hence, the statistically insignificant parameters and the parameter interactions are excluded in the modeled second degree polynomial Eq. (32). In this analysis the linear terms A, B, C and E are found to be significant. Among parameter square terms only ${C}^{2}$ is significant and among all the ten two-parameter interaction combinations only AE, BC, BE and CE are found to be significant. Here A, B, C, D and E represent the GO nanoparticle concentration, MoS_2_ nanoparticle concentration, mass of the nanofluid inserted inside the heat pipe ${(\widehat{M}}_{hnl}),$ rotation speed of the heat pipe $(\Omega )$ and the temperature difference between the adiabatic zone and condenser zone of the heat pipe respectively. The coefficients of different terms in the fitted regression model (32) are given in Table 6.

**Table 5:** Table for significant and insignificant parameters.

Source	DF	Adj SS	Adj MS	*F*-Value	*P*-Value	Comment
Model	20	8047.99	402.40	842.39	0.000	*
Linear	5	7831.14	1566.23	3278.77	0.000	*
A	1	72.36	72.36	151.48	0.000	Significant
B	1	234.31	234.31	490.52	0.000	Significant
C	1	1576.89	1576.89	3301.10	0.000	Significant
D	1	1.06	1.06	2.21	0.165	Insignificant
E	1	5946.51	5946.51	12,448.56	0.000	Significant
Square	5	63.34	12.67	26.52	0.000	*
A*A	1	0.00	0.00	0.00	0.995	Insignificant
B*B	1	0.06	0.06	0.13	0.729	Insignificant
C*C	1	61.58	61.58	128.91	0.000	Significant
D*D	1	0.01	0.01	0.02	0.878	Insignificant
E*E	1	0.18	0.18	0.39	0.547	Insignificant
2-Way Interaction	10	153.51	15.35	32.14	0.000	*
A*B	1	0.75	0.75	1.57	0.236	Insignificant
A*C	1	1.98	1.98	4.14	0.067	Insignificant
A*D	1	1.00	1.00	2.10	0.176	Insignificant
A*E	1	6.52	6.52	13.65	0.004	Significant
B*C	1	5.63	5.63	11.79	0.006	Significant
B*D	1	0.52	0.52	1.10	0.317	Insignificant
B*E	1	19.38	19.38	40.57	0.000	Significant
C*D	1	0.71	0.71	1.49	0.248	Insignificant
C*E	1	116.57	116.57	244.03	0.000	Significant
D*E	1	0.44	0.44	0.92	0.357	Insignificant
Error	11	5.25	0.48			
Lack-of-Fit	6	5.25	0.88	*	*	
Pure Error	5	0.00	0.00			
Total	31	8053.24

**Table 6:** Coded Coefficients for fitted quadratic regression model.

Term	Coef	SE Coef	*T*-Value	*P*-Value	VIF	Comment
Constant	46.610	0.276	169.08	0.000		*
A	1.736	0.141	12.31	0.000	1.00	Significant
B	3.125	0.141	22.15	0.000	1.00	Significant
C	− 8.106	0.141	− 57.46	0.000	1.00	Significant
D	− 0.210	0.141	− 1.49	0.165	1.00	Insignificant
E	15.741	0.141	111.57	0.000	1.00	Significant
A*A	− 0.001	0.128	− 0.01	0.995	1.02	Insignificant
B*B	0.045	0.128	0.36	0.729	1.02	Insignificant
C*C	1.449	0.128	11.35	0.000	1.02	Significant
D*D	0.020	0.128	0.16	0.878	1.02	Insignificant
E*E	− 0.079	0.128	− 0.62	0.547	1.02	Insignificant
A*B	0.216	0.173	1.25	0.236	1.00	Insignificant
A*C	− 0.351	0.173	− 2.03	0.067	1.00	Insignificant
A*D	− 0.250	0.173	− 1.45	0.176	1.00	Insignificant
A*E	0.638	0.173	3.69	0.004	1.00	Significant
B*C	− 0.593	0.173	− 3.43	0.006	1.00	Significant
B*D	− 0.181	0.173	− 1.05	0.317	1.00	Insignificant
B*E	1.101	0.173	6.37	0.000	1.00	Significant
C*D	0.211	0.173	1.22	0.248	1.00	Insignificant
C*E	− 2.699	0.173	− 15.62	0.000	1.00	Significant
D*E	− 0.166	0.173	− 0.96	0.357	1.00	Insignificant

### Goodness of fitted model and error interpretations

The accuracy of the model is determined by computing the coefficient of determination $({R}^{2})$, which is found to be 0.9993. The closeness of this value to 1 (unity) proves the goodness of fitted model. To check the effectiveness of the model to predict the heat transfer outputs for new values of input parameters, the predicted ${R}^{2}$ is also calculated and found to be 0.9825, which means the fitted model is 98.25% effective to predict the accurate output.

**Figure 10:**
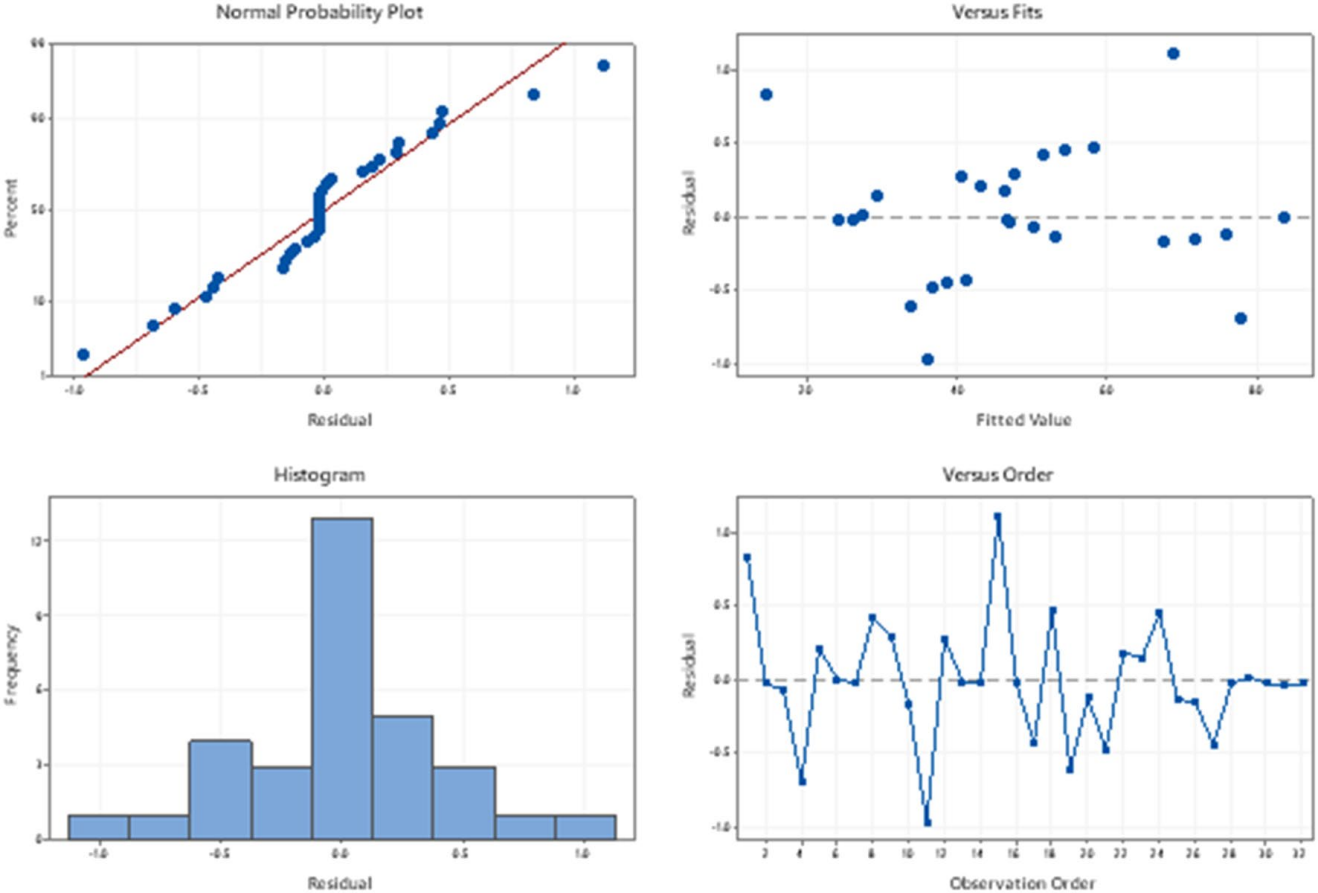
Residual plots for heat transfer.

To confirm the adequacy of this analysis different plots are shown in Fig. 10. The residual have been plotted and the necessary residual assumptions for analysis of variance are checked. The normal probability plot of residuals indicates all the residuals are closed to the straight line without major deviations, which confirms that the residuals are normally distributed. The same is also depicted from the residual histogram. The randomness in the Residual versus fit plot and residual versus scatter plot also confirms the constant variance and independent nature of these residuals.

### Response surface interpretations: (need to be written carefully)

**Figure 11:**
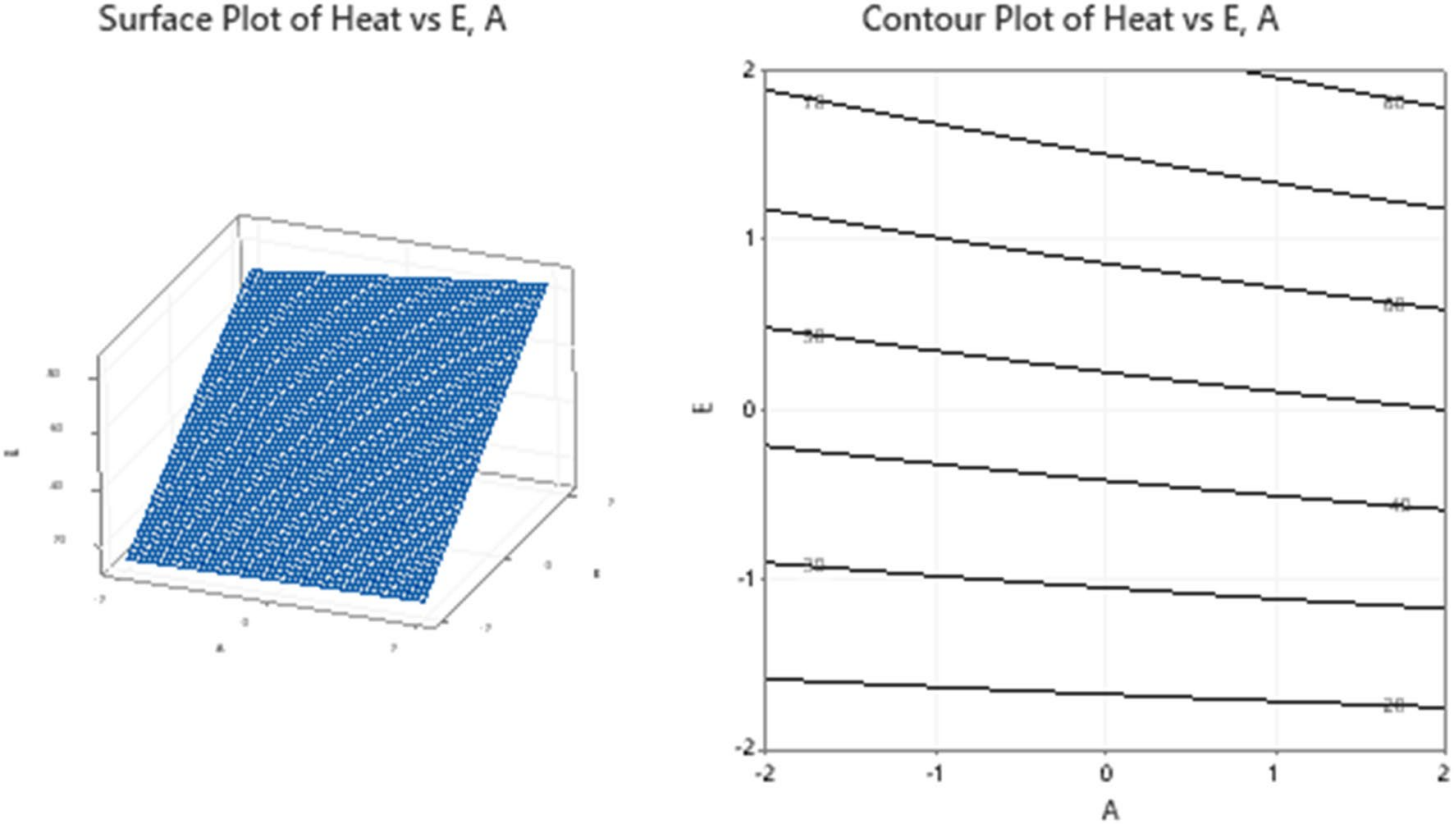
Plots for Heat Vs A, E keeping B, C and D at middle level.

**Figure 12:**
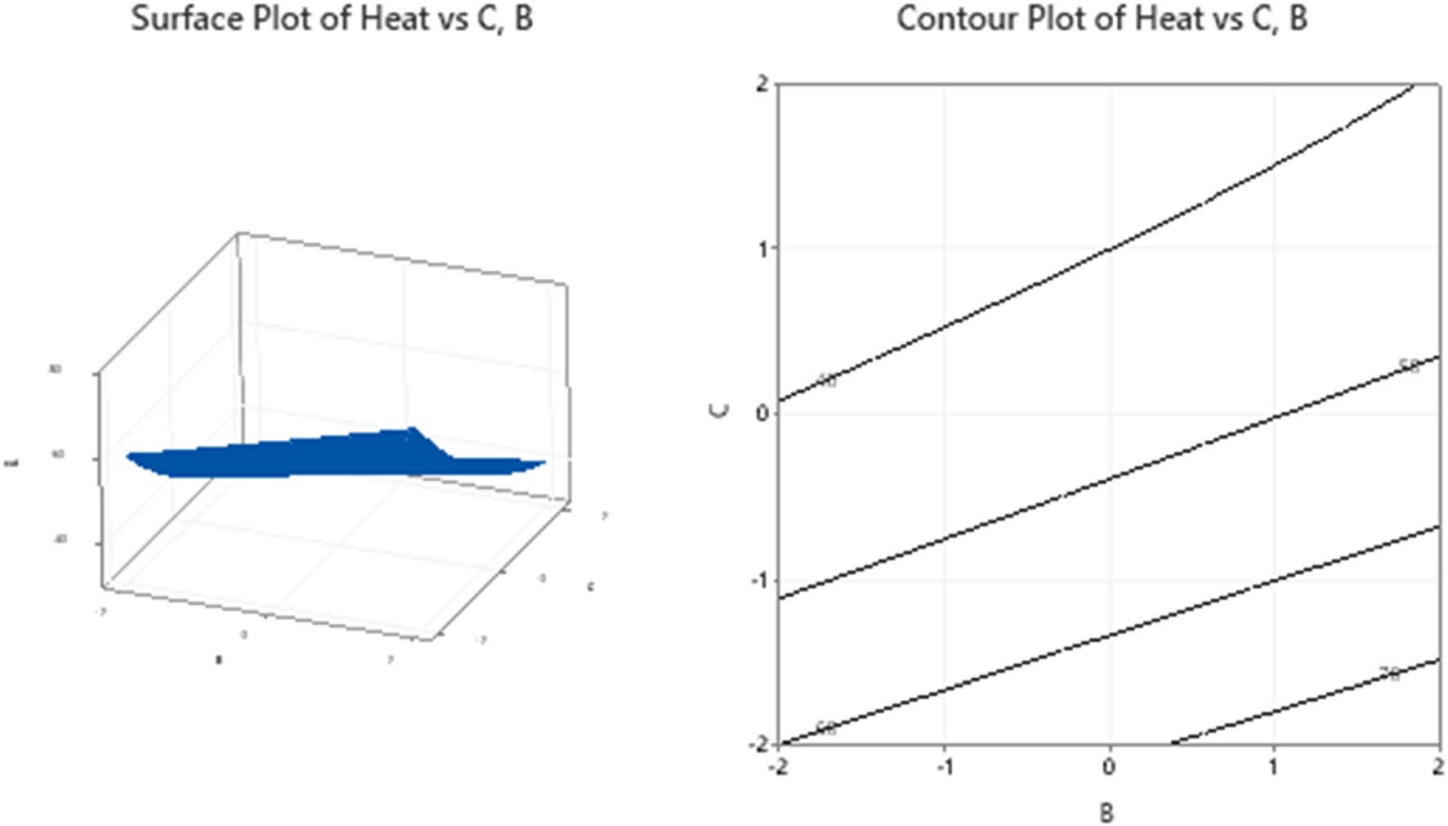
Plots for Heat Vs B, C keeping A, D and E at middle level.

**Figure 13:**
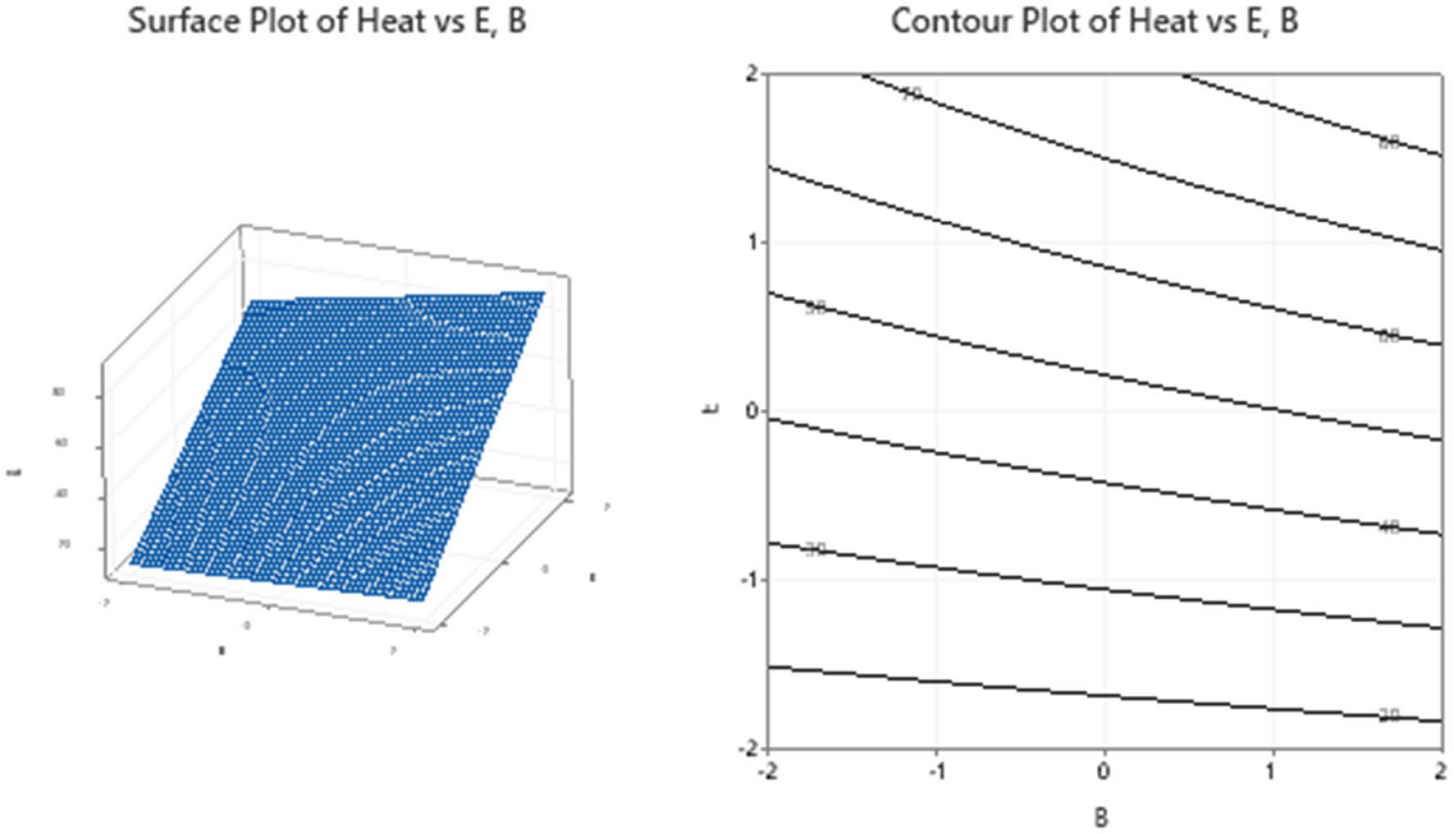
Plots for Heat Vs B, E keeping A, C and D at middle level.

**Figure 14:**
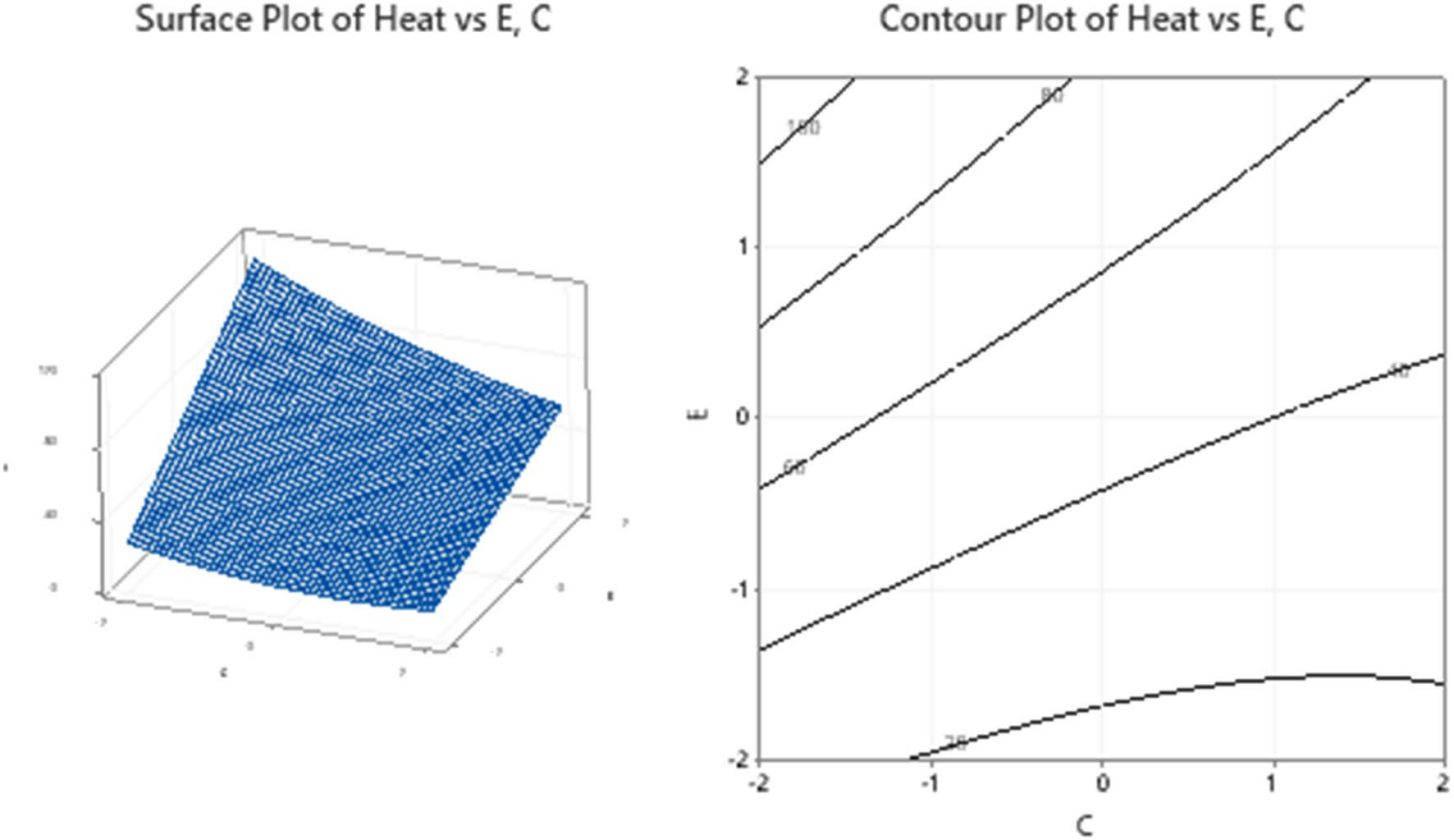
Plots for Heat Vs C, E keeping A, B and D at middle level.

The surfaces for different combinations of significant response variables and the corresponding contour plots are created and shown in Figs. 11, 12, 13 and 14. The monotonically increasing nature of the surface in Fig. 11, depicts that the heat transfer is maximum for higher level values of A and E, while the other variables B, C, and D are kept at the middle level. The symmetry of contour plot also signifies the increase in heat transfer with increasing A and E values. In Fig. 12, the surface for Heat Vs B, C and the corresponding contours show the maximum heat transfer for higher level value of B and lower level value of C, but this effect is not linear in nature.

The surfaces and contours in Fig. 13, depict the occurrence of maximum heat transfer for higher level values for both B and E. As compared to E axis, the surface has a smaller slop with B axis, explains the higher dependence of heat transfer on temperature difference as compared to the nanoparticle concentration. The dependence of inlet mass and temperature difference on the overall heat transfer is shown in Fig. 14. The surface plot and the corresponding contours demonstrate that the heat transfer is more dependent on temperature difference than the total inlet fluid mass.

## Sensitivity study

To check the sensitivity of heat transfer over the input parameters, viz. nanoparticle concentrations, mass of the fluid, rotation speed of the heat pipe et cetera, one variable at a time approach, in combination with partial derivatives is considered. The following sensitivity functions are constructed using Eq. (32).

33 $${\varvec{S}}\left( {{\varvec{Q}}/{\varvec{A}}} \right) = \frac{{\partial {\varvec{Q}}}}{{\partial {\varvec{A}}}} = 1.736 + 0.638\user2{ E}$$

34 $${\varvec{S}}\left( {{\varvec{Q}}/{\varvec{B}}} \right) = \frac{{\partial {\varvec{Q}}}}{{\partial {\varvec{B}}}} = 3.125 - 0.593{\varvec{C}} + 1.101{\varvec{E}}$$

35 $${\varvec{S}}\left( {{\varvec{Q}}/{\varvec{C}}} \right) = \frac{{\partial {\varvec{Q}}}}{{\partial {\varvec{C}}}} = - 8.106 + 2.898\user2{ C} - 0.593\user2{ B} - 2.699\user2{ E}$$

36 $${\varvec{S}}\left( {{\varvec{Q}}/{\varvec{E}}} \right) = \frac{{\partial {\varvec{Q}}}}{{\partial {\varvec{E}}}} = 15.741 + 0.638\user2{ A} + 1.101\user2{ B} - 2.699\user2{ C}$$

**Figure 15:**
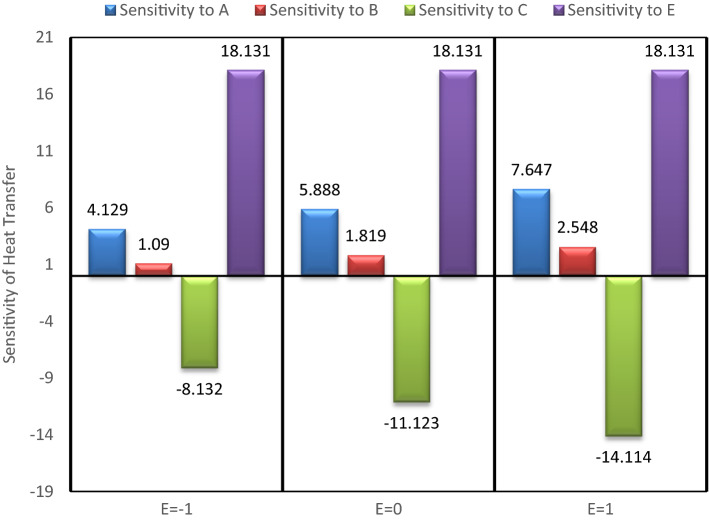
Sensitivity plot for A =  − 1, C =  − 1 at B = 0.

**Figure 16:**
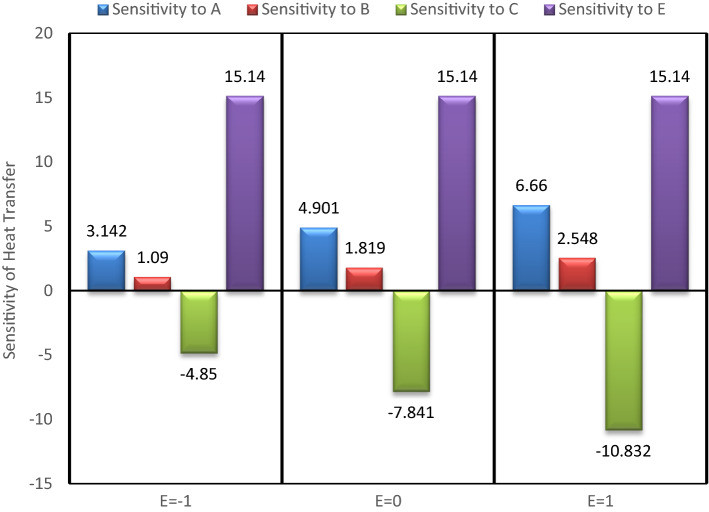
Sensitivity plot for A =  − 1, C = 0 at B = 0.

**Figure 17:**
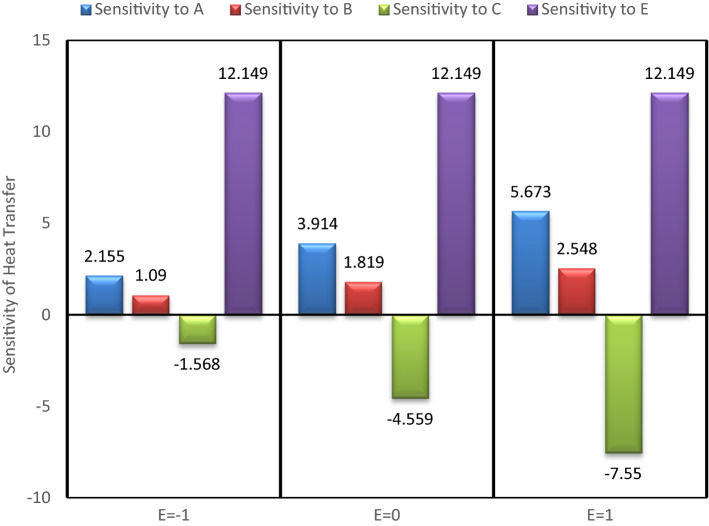
Sensitivity plot for A =  − 1, C = 1 at B = 0.

**Figure 18:**
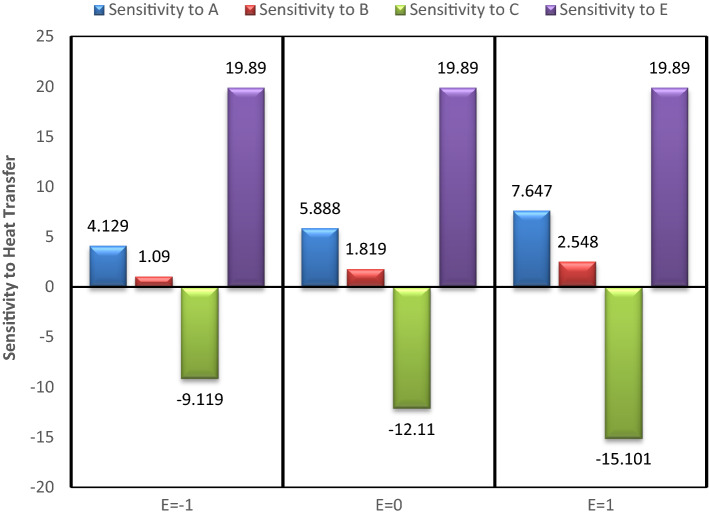
Sensitivity plot for A = 0, C =  − 1 at B = 0.

In the present analysis, to check the sensitivity of heat transfer on different input variable, B variable is kept fixed at middle value (0), and changing the values of other variables values from −1, 0 to 1. The sensitivity bar plots are shown in Figs. 15, 16, 17 and 18.

The increasing heat transfer is shown by upward direction bar, while decreasing heat transfer is presented as downward bar. The height of the bars represents the magnitude of sensitivity. The output (Heat transfer) is more sensitive to the input variable for which the bar is highest. As the bars corresponding to the A, B and E are always in positive direction, therefore the nanoparticle concentrations (A & B) and the temperature difference (E) are helpful in increasing the heat transfer. The negative bar for C represents the decreasing behaviour of heat transfer with increased inlet mass of the fluid (C). From Figs. 15, 16, 17 and 18 it is clear that the heat transfer is most positively sensitive to the temperature difference and negatively sensitive to the fluid mass (C). Figure 15 depicts that the sensitivity to E remains constant for all the possible values of E, i.e. −1, 0 and 1. The same trend is also observed in all the sensitivity plots (Figs. 15, 16, 17 and 18). This means that keeping B at middle value, the sensitivity to E is independent of the variable E, and it can be concluded that here is no interaction of E (temperature difference) with itself.

As observed from then sensitivity trends for A and B, the sensitivity of heat transfer on GO and MoS_2_ nanoparticle concentrations has an increasing effect for increasing values of E. The negative sensitivity to C also increases with increasing values of E. From these trends it is inferred that the nanoparticles concentration and the temperature difference have positive interactions while the mass of the fluid and the temperature difference have negative interactions effects. After the temperature difference, GO nanoparticle concentration is found to be the most sensitive parameter for the enhanced heat transfer.

## Conclusion

In this theoretical work, a sensitivity analysis for heat transfer through the rotating heat pipe with hybrid nanofluid is presented. The impact of rotating heat pipe parameters; hybrid nano particle concentrations, rotation speed, temperature difference, and inlet mass on the liquid film evolution and heat transfer are discussed. The paper reveals the following outcomes:

- Using hybrid nanoparticles with the working fluid of the heat pipe rises its total heat transfer rate.
- Rising the inlet mass to the heat pipe reduces the total heat transfer and increases the difference of the liquid film thickness and reduces the total heat transfer.
- The impact of increasing the volume fraction of GO on the total heat transfer and liquid film thickness is greater than that of MoS_2_.
- Increasing the rotation speed rises the difference of the liquid film thickness between the evaporator and condenser but it slightly influences the heat transfer.
- Increasing the temperature difference between the evaporator and condenser by about 20 °C rises the heat transfer about 62 and the liquid film thickness between them about 0.078 × 10^−4^.
- The heat transfer enhancement is more sensitive to GO nanoparticles as compared to MoS_2_ nanoparticles.
- Both the nanoparticles (GO and MoS_2_), and the temperature difference show the positive sensitivity on total heat transfer, whereas the inlet hybrid nanofluid mass shows the negative sensitivity.

## Data Availability

All data generated and analysed during this study are included in this article.
